# Systematic QM/MM Study for Predicting ^31^P NMR Chemical Shifts of Adenosine Nucleotides in Solution and Stages of ATP Hydrolysis in a Protein Environment

**DOI:** 10.1021/acs.jctc.3c01280

**Published:** 2024-03-18

**Authors:** Judit Katalin Szántó, Johannes C. B. Dietschreit, Mikhail Shein, Anne K. Schütz, Christian Ochsenfeld

**Affiliations:** † Chair of Theoretical Chemistry, Department of Chemistry, University of Munich (LMU), Butenandtstr. 7, D-81377 München, Germany; ‡ Department of Chemistry, University of Munich (LMU), Butenandtstr. 5-13, D-81377 München, Germany; § Max Planck Institute for Solid State Research, Heisenbergstr. 1, D-70569 Stuttgart, Germany; ∥ Department of Materials Science and Engineering, Massachusetts Institute of Technology, Cambridge, Massachusetts 02139, United States

## Abstract

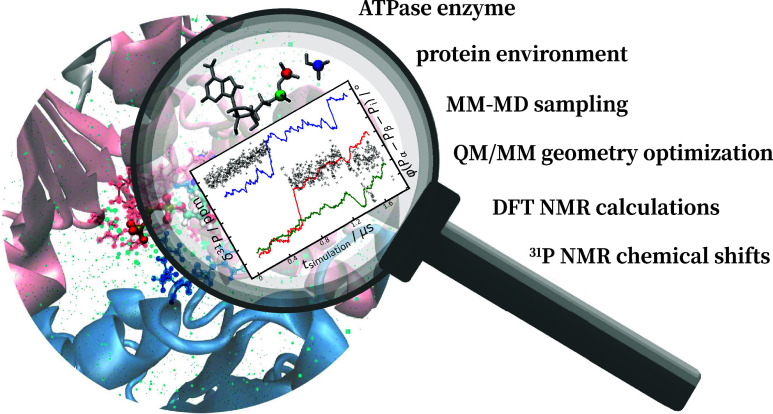

NMR (nuclear magnetic resonance) spectroscopy allows for important atomistic insights into the structure and dynamics of biological macromolecules; however, reliable assignments of experimental spectra are often difficult. Herein, quantum mechanical/molecular mechanical (QM/MM) calculations can provide crucial support. A major problem for the simulations is that experimental NMR signals are time-averaged over much longer time scales, and since computed chemical shifts are highly sensitive to local changes in the electronic and structural environment, sufficiently large averages over representative structural ensembles are essential. This entails high computational demands for reliable simulations. For NMR measurements in biological systems, a nucleus of major interest is ^31^P since it is both highly present (e.g., in nucleic acids) and easily observable. The focus of our present study is to develop a robust and computationally cost-efficient framework for simulating ^31^P NMR chemical shifts of nucleotides. We apply this scheme to study the different stages of the ATP hydrolysis reaction catalyzed by p97. Our methodology is based on MM molecular dynamics (MM-MD) sampling, followed by QM/MM structure optimizations and NMR calculations. Overall, our study is one of the most comprehensive QM-based ^31^P studies in a protein environment and the first to provide computed NMR chemical shifts for multiple nucleotide states in a protein environment. This study sheds light on a process that is challenging to probe experimentally and aims to bridge the gap between measured and calculated NMR spectroscopic properties.

## 1 Introduction

Phosphate ester hydrolysis is one of the most important biochemical transformations and the most frequent chemical reaction occurring in the human body.^1^ As adenosine triphosphate (ATP) undergoes hydrolysis to adenosine diphosphate (ADP) and inorganic phosphate (P_i_), energy is released that can be used for various cellular processes, making ATP the primary energy currency of cells. In general, ATP hydrolysis has been the subject of many experimental and computational studies that provided unique insights into the mechanism and the energetic landscape of the catalytic reaction.^2−4^ The question of whether hydrolysis follows an associative, dissociative, or concerted pathway has sparked controversial debates in the literature. Despite numerous quantum mechanical/molecular mechanical (QM/MM) free energy studies on the general mechanism of phosphor ester hydrolysis, the question of whether ATP hydrolysis proceeds through the formation of short- or long-lived intermediates or no intermediates at all remains unanswered.^4−18^ Reaction intermediates were postulated for ATPases many decades ago,^19,20^ but escaped experimental observation for a long time. Recently, the focus of experimental studies became to capture intermediate states within the ATP hydrolysis cycle.^21,22^ In human ATPase p97, monitoring the enzymatic activity via real-time nuclear magnetic resonance (NMR) led to the observation of a reaction intermediate with a lifetime of approximately 1 min.^23^ Overall, free energy calculation studies and experimental NMR investigations highlight numerous unanswered questions regarding phosphate hydrolysis in diverse catalytic environments. To the best of our knowledge, there is no QM/MM free energy study answering these questions for the ATP hydrolysis reaction catalyzed by p97. Therefore, in order to resolve some of these questions, the aim of our study is to compute chemical shifts in different stages of the phosphate hydrolysis process catalyzed by p97 and to understand local structures in light of experimentally measured NMR shifts.

NMR is a powerful experimental method to investigate protein structure and dynamics.^24^ Recently, the focus of protein NMR has shifted from structure determination toward probing protein motions on multiple time scales and relating them to function.^25^ For the NMR spectroscopy of biomolecules, phosphorus has gained increasing interest lately, due to the occurrence of this element in nucleic acids, lipids, and nucleotide substrates.^26−28^ The ^31^P nucleus is of particular interest to experimental and theoretical NMR studies because of its favorable properties: it has a spin of 1/2, 100% natural abundance, moderate relaxation times, and a high gyromagnetic ratio. Additionally, like other heavy nuclei, ^31^P covers a wide range of chemical shifts of more than 600 ppm rendering it an excellent probe of its chemical environment.^29^ For example, ^31^P chemical shifts provide valuable structural information about the sugar–phosphate backbone in nucleic acids,^30^ since they serve as direct probes of phosphate ester torsional angles and O–P–O bond angles along the anhydride backbone, which largely define the conformation of nucleic acids.

To understand experimental NMR spectroscopic data, QM calculations of chemical shifts are highly valuable. Combined theoretical and experimental studies on ^31^P NMR helped, e.g., to understand the thiophosphorylation of amino acids^31^ and the impact of the backbone torsion angles on J-coupling constants in nucleic acids.^32^ Variations in certain torsion angles can lead to changes of 6 ppm in the ^31^P chemical shifts.^33^ Thus, theoretical studies can couple structural motifs to NMR observables and aid the assignment of NMR resonances. A major challenge is the different time scales: the recording of a single NMR signal (the free induction decay) takes milliseconds to seconds, whereas chemical reactions occur typically on a much shorter time scale. In contrast, QM calculations are often performed on single configurations, which are fleeting snapshots of the molecular system. Therefore, proper comparison of experimental measurements and QM calculations requires averaging over a representative structural ensemble for the latter, especially since computed NMR chemical shifts are highly sensitive to local changes in the molecular structure. Even small variations in bond lengths and angles can have a profound impact on the computed shielding values.^33,34^ Hence, for theoretical studies of biomolecular systems, the combination of at least the density functional theory (DFT) level and molecular dynamics (MD) would be desirable. However, such simulations are very costly. Therefore, one often reverts to the simpler force field approximations (MM-MD) that offer valuable insights into the dynamic behavior and conformational changes of biomolecules. While the combination of MM-MD with DFT NMR calculations^33,35−38^ offers significant improvements compared with methods relying on static structures of biomolecules, deficiencies in the structural description at the MM level can cause severe problems for subsequent property calculations at the QM level. Furthermore, the QM-based prediction of NMR properties in nucleic acid phosphates can be challenging because phosphate groups represent structurally the most variable segment of nucleic acids.^39^ Several studies on nucleic acids^40,41^ and proteins^36,42−44^ have highlighted the importance of combining the QM-based prediction of chemical shifts with MM-MD simulations to account for conformational diversity and solvent effects. As mentioned above, NMR shift computations are further complicated by the poor description of phosphate structures by classical force fields. For example, a study combining MM-MD sampling and DFT NMR calculations on ^31^P chemical shifts of a B-DNA sequence^45^ suggests that bond lengths sampled by classical MD are likely unrealistic, a problem that was also reported in a benchmark study of ^31^P NMR parameters.^40^ The general importance of a correct local structure for spectroscopic properties has also been shown by Vogler et al.^46^ with respect to hyperfine coupling constants, which are equally sensitive as NMR shieldings.

Besides accounting for a structural ensemble, explicit solvation is often crucial. Neglecting solvation effects, particularly for charged molecules like nucleotides, can lead to wrong electronic structures.^47^ Furthermore, previous studies^40,48^ suggest that the mobility of the solvent has a drastic impact on the NMR parameters of nucleic acid phosphates, which highlights the necessity of explicitly treating phosphate–water interactions. Therefore, explicit solvent molecules, especially in the first solvation shell around the phosphate group^33^ should be treated quantum mechanically when modeling such systems. Additionally, ions within the solvent are important, especially the cations. A study by Benda et al.^48^ investigated ^31^P shielding tensors in the nucleic acid backbone and their dependence on coordinating Mg^2+^ ions. Their findings highlight the significance of local Mg^2+^ coordination, which can lead to changes of up to 9 ppm in the computed ^31^P chemical shifts. Divalent cations are often present and sometimes are even involved in catalytic mechanisms. A bound Mg^2+^ cofactor is a key common feature among nucleotide hydrolases and the presence of metal ions, e.g., Mg^2+^, Mn^2+^, Zn^2+^, or Fe^2+/3+^, in the active site can also change the catalytic effect,^6,7,49,50^ posing challenges in the exploration of reaction pathways across diverse catalytic environments.

This study is organized as follows: first, we outline the computational setup, followed by our findings, with a focus on correlations between structure and computed NMR chemical shifts. In order to understand deviations between computed and measured chemical shifts, we compare downfield- vs upfield-shifted populations and apply a classification algorithm.

## 2 Methods

In this study, we have developed a robust methodology designed to address limitations and pitfalls encountered in the literature when predicting NMR chemical shifts, as outlined in the previous section. Owing to the much higher computational cost of QM-MD simulations, our pragmatic approach to tackling these challenges can be summarized as follows:

1. MM-MD sampling of the nucleotide in solution and inside the enzyme creating a conformational ensemble. MM-MD includes explicit solvent and divalent ions around the nucleotides, and thus accounts for important nucleotide–environment interactions.
2. QM/MM optimizations of MM-MD snapshots are performed to counteract force field deficiencies, while comprising with respect to the ensemble sampling.
3. We ensure QM size convergence in the NMR calculations.
4. Detailed analysis of calculated shifts pinpoints structural features that are specific for downfield- vs upfield-shifted populations.

Before carrying out QM/MM calculations in the protein environment, we refined our setup on chemical shifts of adenosine di- and triphosphates in solution and ensured agreement with experimentally observed values.

### 2.1 MD Setup

MM-MD trajectories of the nucleotide-bound states in p97 ATPase (pre- and posthydrolysis states and the postulated intermediate) have been provided by the authors of ref (56), which are based on the following X-ray structures (Table 1).

**Table 1:** PDB ID of the X-ray Structures Used as Starting Structures for the MD Trajectories of the Nucleotide-Bound States in p97

	PDB ID	modifications
ATP	4KO8(51)	ATPγS was converted to ATP
ADP.P_i_	4KO8(51)	ATPγS was converted to ADP.P_i_
ADP	1E32(52)	-

The MM-MD simulations of ATP and ADP in solution were carried out using the NAMD software.^53^ The system setup was performed with AmberTools16.^54^ ATP and ADP were described with the parameters from Meagher et al.^55^ for consistency with the p97 simulations^56^ and the nucleotides were solvated in a cubic box of TIP3P water. In order to investigate the influence of the ionic concentration, we carried out simulations with a minimal ionic concentration that just ensured neutral net charge as well as simulations in a high salt buffer, including Na^+^, Mg^2+^, K^+^, and Cl^–^ ions. Trajectories of solvated nucleotides and nucleotides bound to the protein capture different time scales. While simulations in the range of a few nanoseconds are typically suitable to resolve the dynamics of nucleotides and their interactions with solvent molecules, a larger time scale in the range of 1–2 μs is needed to represent the environment of the nucleotides at the active site. For the complete computational details of the MD simulations in solution, see Section S1 of the Supporting Information (SI).

### 2.2 DFT NMR Computation

For every system investigated in this study, the QM size convergence was tested,^57^ i.e., to attain reliable shielding values, a QM region around the nucleus of interest was selected so large that the addition of more atoms would not affect the computed NMR shieldings (for detailed information, see Section S8 of the SI).

The QM region used in the NMR calculations comprises the nucleotide of interest (i.e., ATP, ADP.P_i_, or ADP) and all ions, solvent molecules, and, if present, protein residues which are found within 3.8 Å around the phosphate backbone and the sugar ring of the nucleotide at any point during the MD simulation. In the case of predicting NMR chemical shifts, proximity is more important than biological function. Therefore, when selecting the QM region, amino acids that play an important role in the hydrolysis process but are further away (*d* > 3.8 Å) from the nucleotide remain in the MM region. When we compute chemical shifts for the nucleotides bound to the active site, the QM region is in the range of 500–700 atoms, including protein residues, ions, and water molecules. For solvated nucleotides, the environment is less dense, and the QM region consists of 200–250 atoms, including only ions and water molecules (see Section S9 of the SI for further details). QM/MM NMR calculations were performed using evenly spaced snapshots extracted from the MD trajectories. Frames were taken every 2 ns from the protein MM-MDs, yielding 540 frames for the ATP-bound state, 900 frames for ATP.Pi, and 480 frames for ADP in p97. In solution, snapshots were extracted using intervals of 1 ns, yielding 100 frames of solvated ATP and ADP molecules.

**Figure 1:**
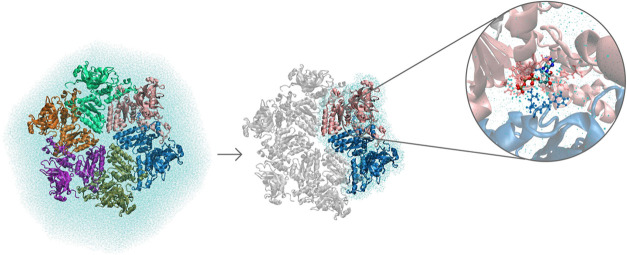
Cutting out two neighboring protein subunits from the solvated hexamer together with 5 Å of solvation shell around the two selected subunits and selecting the QM region. The six subunits are shown as cartoon diagrams in different colors, and the nucleotide of interest (colored by atom type) and amino acids (colored based on which protomer they belong to) in the QM region are represented by sticks. The PDB ID of the X-ray structure is 4KO8.

The p97 enzyme complex has a hexameric structure, where the active sites are located at the interface of the subunits. Therefore, two neighboring protein subunits (ca. 30,000 atoms) were cut out from the full protein hexamer, see Figure 1, where we included both Walker A and B motifs, as well as the arginine fingers, which are responsible for nucleotide binding and hydrolysis,^58^ respectively.

Instead of breaking peptide bonds and cutting through polar bonds between the C and N atoms, the QM/MM boundary was placed such that only nonpolar C–C bonds were cut. In this way, we avoid spurious polarization at the QM/MM boundary.^59^ If single amino acids were included in the QM region, link atoms were introduced between the C_β_ and C_α_ atoms to saturate the QM region using a distance of 1.09 Å. If a series of neighboring amino acids was selected to be treated at the QM level, the bond between the C_α_ and the C_carbonyl_ atoms of the protein backbone was cut (for further details see Section S6 of the SI). After defining the “static” QM region, we employed an automatic workflow that placed the H atoms as links between the MM and QM regions and calculated the charge for all snapshots extracted from the trajectory based on the charge of the central nucleotide (i.e., ATP, ADP.P_i_, or ADP), selected protein residues, and ions.

All NMR calculations were conducted with the program package FermiONs++(60−64) in combination with the LibXC^65^ library of exchange-correlation functionals and OpenMM^66,67^ libraries. The QM/MM interactions are described in an additive scheme using electrostatic embedding. QM/MM NMR calculations were carried out at the B97–2/pcSseg-2^68,69^ level of theory. Jensen and co-workers developed highly successful basis sets^70^ for computing NMR shielding and J-coupling constants,^40,48^ from which we chose the segmented contracted double-ζ basis set (pcSseg-2), as it was optimized for nuclear magnetic shieldings.

Chemical shifts often exhibit fairly low sensitivity to the selection of the exchange-correlation functional,^65,71^ with excellent performance of the KT2 method^72^ for NMR shifts predicted in organophosphorus compounds.^73^ Previous studies have shown that the KT2 and B97–2 functionals yield very comparable NMR shieldings.^36^ In this study, we chose the more expensive B97–2 hybrid GGA functional since it provides greater stability when dealing with negatively charged species due to the inclusion of HartreeFock exchange. QM calculations are sped up by using seminumerical exact exchange (sn-LinK),^74−76^ recently developed in our group, enabling highly efficient hybrid-DFT applications on extended biomolecular systems. For further details about settings used in the DFT NMR calculations, see Section S4 of the Supporting Information.

### 2.3 Choice of Reference

The calculated NMR results are, at first, absolute magnetic shieldings. Therefore, the choice of the referencing method is critical for the interpretation of the computed chemical shifts. ^31^P chemical shift calculations suffer from referencing issues as the standard experimental reference compound (85% aqueous phosphoric acid) is difficult to model.^77,78^ Previous studies have explored and implemented various alternative referencing schemes,^40,44^ revealing that the choice of the referencing approach can dramatically impact the agreement with experimental data. However, in contrast to using a reference compound computed at the same level of theory as the atom of interest, relative referencing within a molecular system compares chemically equivalent nuclei, reduces systematic errors, and outperforms NMR reference schemes that utilize H_3_PO_4_ or PH_3_ as reference compounds. In our study for comparison with the experiment, we defined internal reference values based on the chemical shifts of the P_α_ nucleus. We chose this nucleus as it is not involved in hydrolysis and its immediate chemical environment changes little between ATP and ADP. For further details about the reference values in the solution and in protein environment, see Sections S5 and S7 in the SI.

### 2.4 DFT Structure Optimization

In order to allow for a reliable description of the configurations sampled by MM-MD, the structures of the nucleotides were optimized prior to the NMR computations. To show the necessity of this step, we also computed the NMR shieldings for several systems directly on the MM structures. The QM/MM structure optimizations were performed at the PBEh-3c/def2SVP^79^ level of theory. In the complex protein environment, we used the DL-Find library^80^ implemented in PyChemShell,^81^ whereas for the solvated nucleotides, we used geomeTRIC,^82^ which is directly connected to the Python interface of FermiONs++. Full details regarding the convergence criteria used in the structure optimizations are given in Section S3 of the SI. The structure of the molecules of interest (i.e., ATP, ADP.P_i_, or ADP) was optimized, whereas all other atoms (in QM or MM region alike) were frozen at their original position. We employed the same QM size of 3.8 Å around the nuclei of interest as that for the NMR calculations. The freezing of the environment was done for two reasons: (i) To speed up the optimization and (ii) to ensure a larger diversity among the minimum energy structures as demanded by the cage spanned by the environment, and completely unconstrained minimizations would likely have led to a reduced sampling effect.

## 3 Results and Discussion

We begin by inspecting chemical shifts from simulations conducted under minimal and high ionic conditions in solution, followed by a discussion on the impact of the QM/MM structure optimization. Building on the insights we acquire from the calculation of chemical shifts in solvated nucleotides, we extend our methodology to analyze nucleotides captured at different stages of phosphate hydrolysis catalyzed by p97.

### 3.1 Nucleotides in Solution

**Figure 2:**
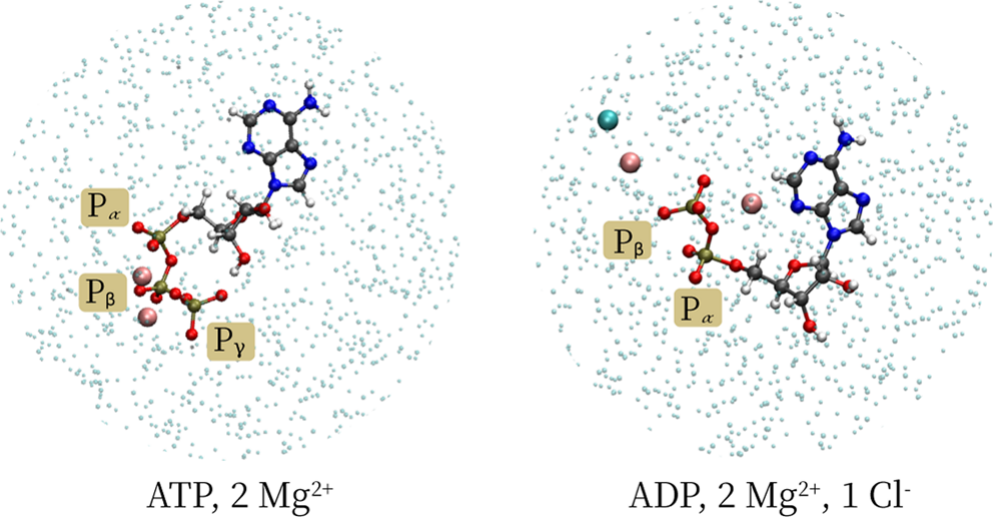
ATP and ADP molecules in solution using minimal ionic concentrations. Atoms of the ATP and ADP molecules are represented in the ball-and-stick mode, Mg^2+^ ions in pink, the Cl^–^ ion as a turquoise sphere, and the atoms of the water molecules as small, transparent spheres.

We begin with simulations in which the nucleotides are solvated in water with just enough counterions to neutralize the charge (from here on referred to as simulations under minimal ionic conditions, Figure 2).

**Figure 3:**
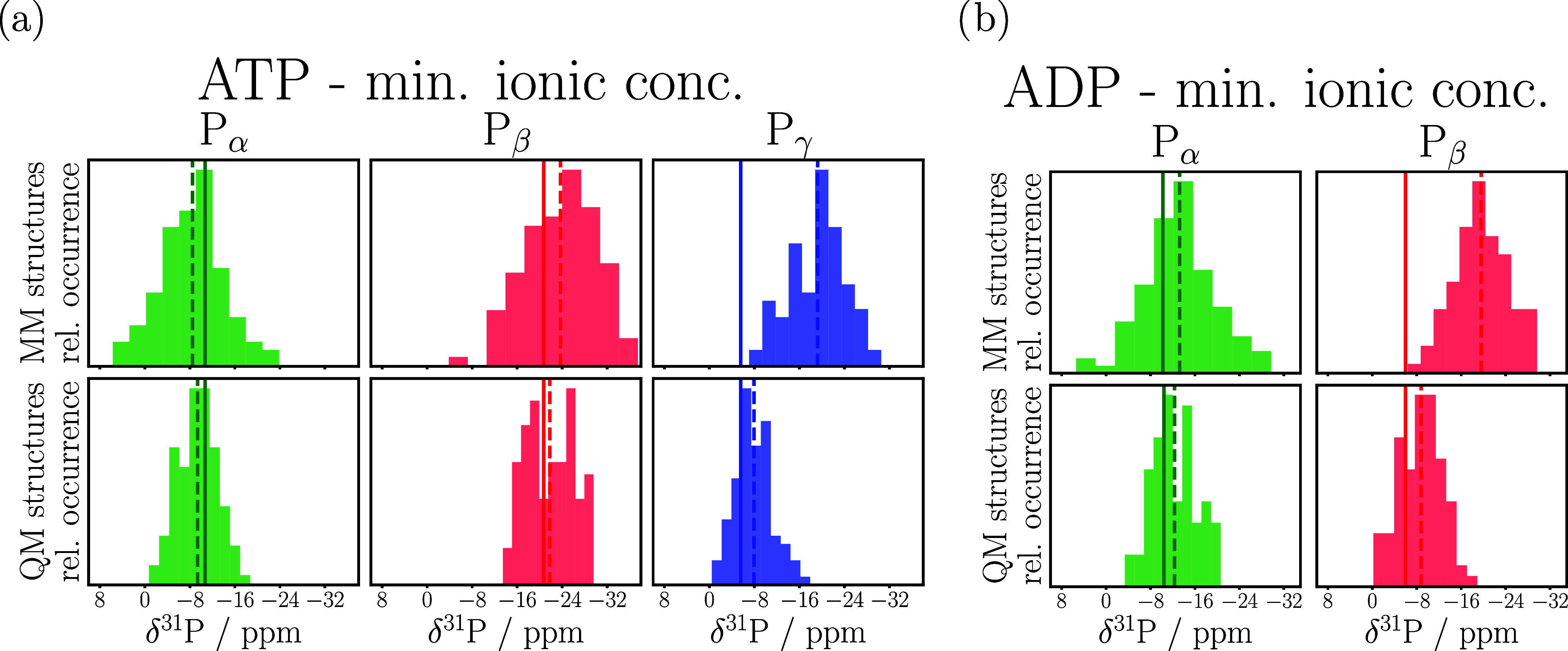
Chemical shifts predicted for the P_α_(green), P_β_(red), and P_γ_(blue) nuclei in MM (top half of the table) and QM geometries (bottom half of the table) of (a) ATP and (b) ADP. The dashed lines mark the mean value of the distributions, and continuous lines represent the experimentally measured values.

The top half of Figure 3 shows the distribution of the NMR chemical shifts predicted from structures taken directly from the MM-MD simulations, whereas the bottom half presents those shifts obtained after the QM/MM structure optimizations. It stands out that chemical shifts predicted from MM structures span a range of 20–30 ppm, while those obtained from QM-optimized structures provide narrower distributions in the range of 15 ppm. This is not unexpected as the constrained minimization not only shifts the bond lengths toward more accurate values but unfortunately removes some of the thermal fluctuations as well.

In general, the best way to compute experimentally measurable observables is via a proper ensemble average, which can be obtained from sufficiently long MD simulations at the highest possible level for the PES (potential energy surface) as a time average. As Figure 3 shows, the averages of the QM/MM NMR shifts obtained directly from the MM-MD simulations are extremely poor. This has to do with the fact that especially the bond lengths sampled by MM-MD are wrong (see Figure 5). Our approach is also motivated by previous studies,^32,34,40,45,48^ which showed that the structure optimization of MM-MD snapshots is a necessary step for enhancing the accuracy of the predicted NMR properties. The best option would be to sample the geometries by means of pure QM- or QM/MM-MD, which is prohibitively expensive due to the size of the system at this stage. An alternative would be to reweight the MM structures by their QM energy to obtain an approximate QM-energy-weighted ensemble average. In our present case, this does not work, since the distribution of bond lengths sampled by the MM force field has basically no overlap with a distribution expected from a QM description. This means that the only way to obtain reasonable geometrical features for the nucleotide is by performing a QM/MM minimization, which partially destroys the ensemble, as it effectively cools structures down to 0 K. Therefore, we try to preserve thermal fluctuations by only minimizing the nucleotide and keeping its environment fixed. Hence, the environment retains the full temperature, and the minimization of the nucleotide is constrained by the fixed orientation of the environment, and thus the thermal fluctuations are partially preserved in the nucleotide. Extremely fast and still accurate QM/MM schemes or replacing the *ab initio* part with high-quality machine-learned interatomic potentials might be options in the future; however, they are currently not available at this scale.

The changes in the chemical shift values before and after QM/MM structure optimization are the most significant for the last P atom of the backbone, e.g., P_γ_ in the case of ATP. Here, we observe a 11 ppm downfield shift (see Figure 3a and Table S6). The same trend holds for results obtained for ADP, the chemical shift of P_β_ changes the most (see Figure 3b and Table S7). This observation also applies to the unreferenced absolute shieldings; hence, the effect cannot be attributed to the referencing scheme. During structure optimization, the positions of the atoms are adjusted to minimize the total energy of the system. As a result, in contrast to atoms closer to the ribose ring, those at the terminus of the phosphate backbone experience greater freedom during structure optimization due to fewer constraining interactions, allowing them to undergo larger deviations from their initial positions. It is important to highlight that comparison with experimental data shows that a refinement of the molecular structure is crucial when predicting NMR chemical shieldings, which depend on subtle stereoelectronic effects.

In addition to the simulations that had the minimal number of counterions, we performed simulations with Na^+^, Mg^2+^, K^+^, and Cl^–^ ions in a higher concentration. We carried out simulations with two different simulation box sizes, one containing a single nucleotide and the other two nucleotides; details on simulations carried out under minimal and high ionic conditions can be found in the Supporting Information, Section S1 “MD procedure for nucleotides in solution.”

Inspection of Tables 2, 3, and 4 shows again that using MM geometries for NMR shift prediction would incur larger errors, since especially the equilibrium bond lengths are too short in the MM force field parameters (see Figure 5). Out of all nuclei, the error is the largest for the last P atom of the backbone, but structure optimization always reduces the average deviation per atom (*P̅*) significantly. For the solvated ADP molecule, both simulations with a high ionic concentration yield NMR shift averages closer to the experimentally measured values. The average deviation of 7.87 ppm from the simulations using a minimal ionic concentration is reduced to 4.80 ppm (single ADP) and 3.55 ppm (two ADP), respectively.

**Table 2:** Deviations in ppm for Predicting ^31^P NMR Chemical Shifts in Solvated ATP and ADP Molecules under Minimal Ionic Conditions^a^

			|δ*P*_calc._ – δ*P*_exp._|/ppm			
			P_α_	P_β_	P_γ_	*P̅*
min. ionic conditions	ATP	MM	2.66	4.19	13.27	6.71
		QM	1.76	2.30	1.95	2.00
	ADP	MM	2.66	13.08	−	7.87
		QM	1.75	2.25	−	2.00

a *P̅* denotes the average deviations.

**Table 3:** Deviations in ppm for Predicting ^31^P NMR Chemical Shifts in Solvated ATP and ADP Molecules in High Ionic Conditions^a^

			|δ*P*_calc._ – δ*P*_exp._|/ppm			
			P_α_	P_β_	P_γ_	*P̅*
high ionic conditions	single ATP	MM	3.91	5.38	8.55	5.95
		QM	0.03	5.11	2.89	2.68
	two ATP	MM	2.14	1.06	8.45	3.88
		QM	0.01	1.03	1.60	0.88
	single ADP	MM	1.38	8.21	−	4.80
		QM	1.18	1.64	−	1.41
	two ADP	MM	1.91	5.19	−	3.55
		*QM*	0.39	2.28	−	1.34

a *P̅* denotes the average deviations.

In the case of the ATP molecule, we see a significant improvement when using higher ionic concentrations for the simulations containing two ATP molecules in the simulation box. Here, the 6.71 ppm average deviation from the minimal ionic concentration simulation is reduced to 3.88 ppm. In contrast, simulations of single ATP in a solution with high ionic concentration (5.95 ppm deviation) yield similar errors to those carried out under minimal ionic conditions (6.71 ppm deviation) for the unoptimized MM structures. Structure optimization reduces the average error to 2.68 ppm but does not solve the core problem, especially for P_β_, where the error remains significantly high compared with other trajectories. This has several reasons. The inspection of the P_α_ – P_β_ – P_γ_ angle reveals two different ATP conformers in solution (see Figure S19). One conformer is characterized by an elongated phosphate tail (observed only in the MD of high ionic conditions + single ATP), whereas the other conformer exhibits a more folded phosphate backbone. While the transition between these two conformers involves relatively minor geometric alterations, previous studies reported energy barriers high enough to hinder the thorough exploration of the configuration space in unbiased molecular simulations.^83,84^

It has been shown that these two ATP configurations are approximately isoenergetic in solution.^83^ However, the Amber force field parameters display a preference for configurations with a folded phosphate tail. Thus, the MM-MD of the single ATP under high ionic conditions samples exclusively configurations from one minimum, as corroborated by the large deviation between calculated and experimentally observed NMR chemical shifts. The QM/MM optimizations do not heal these problems as they merely optimize the structure into the closest local minimum, they only change the P_α_ – P_β_ – P_γ_ by a few degrees (see Table S15), but the minimization will not turn the entire backbone around to the other minimum (for more details, see Supporting Information, Section S11). Further details about sample preparation and NMR measurements can be found in Section S2 of the SI.

### 3.2 Effect of QM/MM Structure Optimization on the Computed NMR Chemical Shifts and P–O Bond Lengths

**Figure 4:**
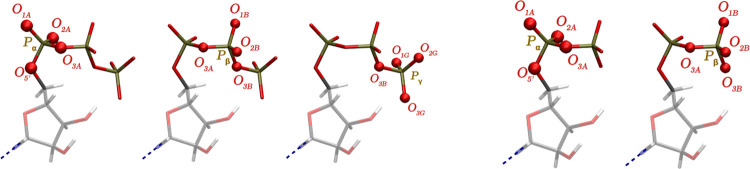
Notation of atoms within the phosphate backbone in the ATP (left) and ADP (right) molecules.

**Figure 5:**
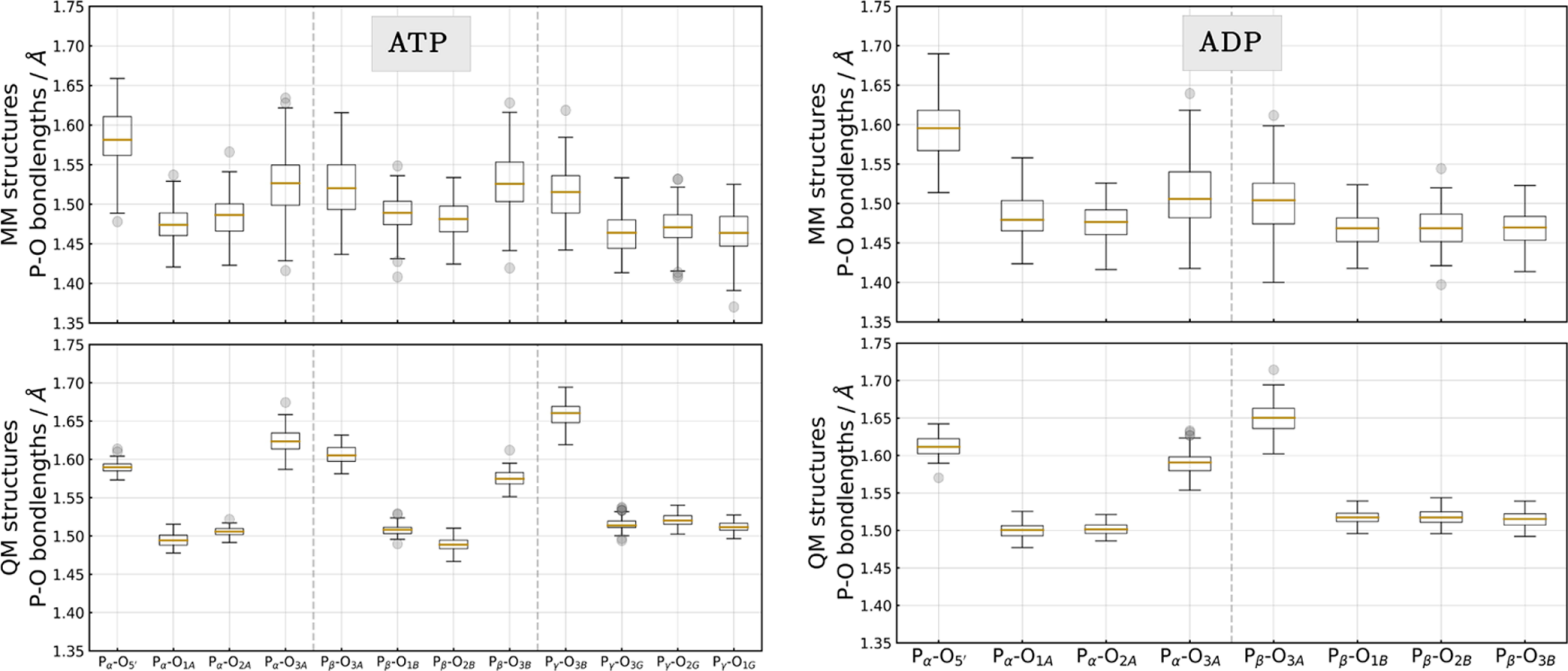
Distribution of the P–O bond lengths in ATP/ADP before (MM structures) and after (QM structures) structure optimization. The boxes show the interquartile range (IQR), the yellow lines represent the median, whiskers extend to 1.5 times the IQR, and outliers are shown as gray dots.

In both ADP and ATP, each phosphate atom is connected to four oxygen atoms (Figure 4). As seen in Figure 3b, after structure optimizations were performed, the distributions of the predicted chemical shifts for the P_β_ nucleus reveal a noticeable downfield shift. From the boxplots in Figure 5, we can see differences between structures directly extracted from the MM-MD trajectories and those after structure optimization. Because of the structure optimization, there is an overall lengthening of the P–O bonds. Notable is the 0.15 Å increase in the P_β_–O_3A_ bond, which can be linked to the downfield shift of the chemical shift computed for the P_β_ nucleus. The same trend holds for the ATP molecule, where the P_γ_–O_3B_ bond changes the most, as well. In the force field ensembles for both ADP and ATP, we see a more uniform distribution of the P–O bonds, and the boxes and whiskers here encompass a broad range, just like the chemical shift distributions (see Figure 3) predicted for the MM snapshots. After structure optimization (QM structures), it becomes apparent that P–O bonds, where oxygen atoms carry a negative charge or form a double bond with the phosphate, are in general shorter by 0.1 Å than the phosphoanhydride P–O bonds along the backbone: P_α_–O_1A_, P_α_–O_2A_, P_β_–O_1B_, P_β_–O_2B_, P_γ_–O_1G_, P_γ_–O_2G_, P_γ_–O_3G_ vs bonds formed with the O_5′_, O_3A_, O_3B_ atoms. This observation will be important later when we look at the anatomy of the postulated intermediate state inside the protein. Together, these results suggest that the observed downfield shifts can be linked to the increased P–O bonds.

### 3.3 Nucleotides Inside the Binding Pocket of p97 ATPase

Building on our insights gained from computing chemical shifts in solution and the QM size convergence study (see Section S8 of the SI), we performed QM/MM structure optimizations and NMR calculations (Table 4) for snapshots extracted from the MD trajectories^56^ started from the crystal structures of p97 (see Table 1).

**Figure 6:**
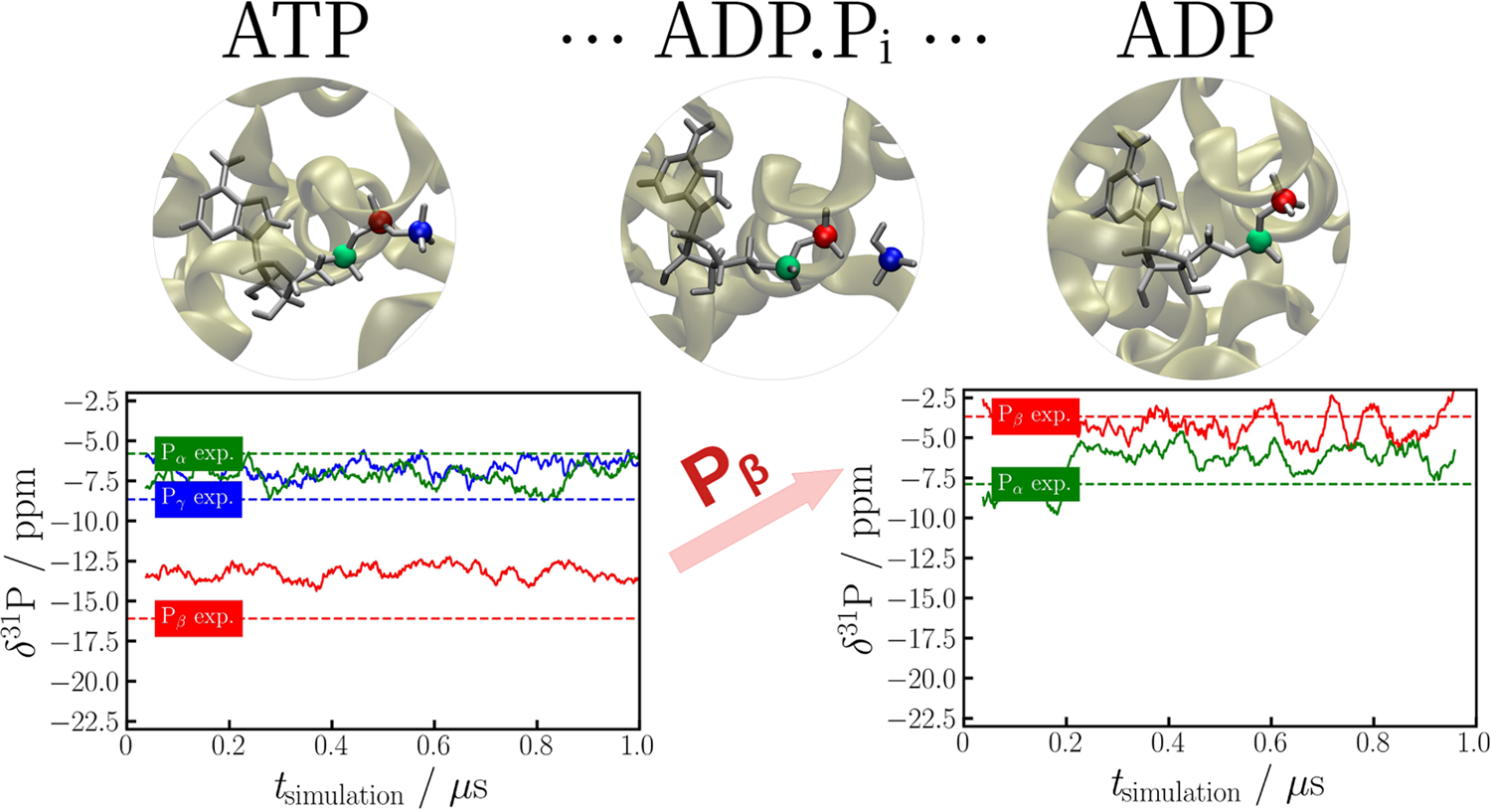
Top: ATP, ADP.P_i_, and ADP inside the binding pocket. Bottom: Time evolution of the ^31^P chemical shifts of the ATP (left) and ADP (right) molecules inside p97. The dashed lines mark experimental values, and continuous lines represent rolling averages from chemical shifts after structure optimization. For the rolling averages, a window size of 20 snapshots was used. The time evolution of chemical shifts corresponding to ADP.P_i_ is shown in Figure 8 (P_β_), Figure S3 (P_α_), and Figure S4 (P_i_).

**Figure 7:**
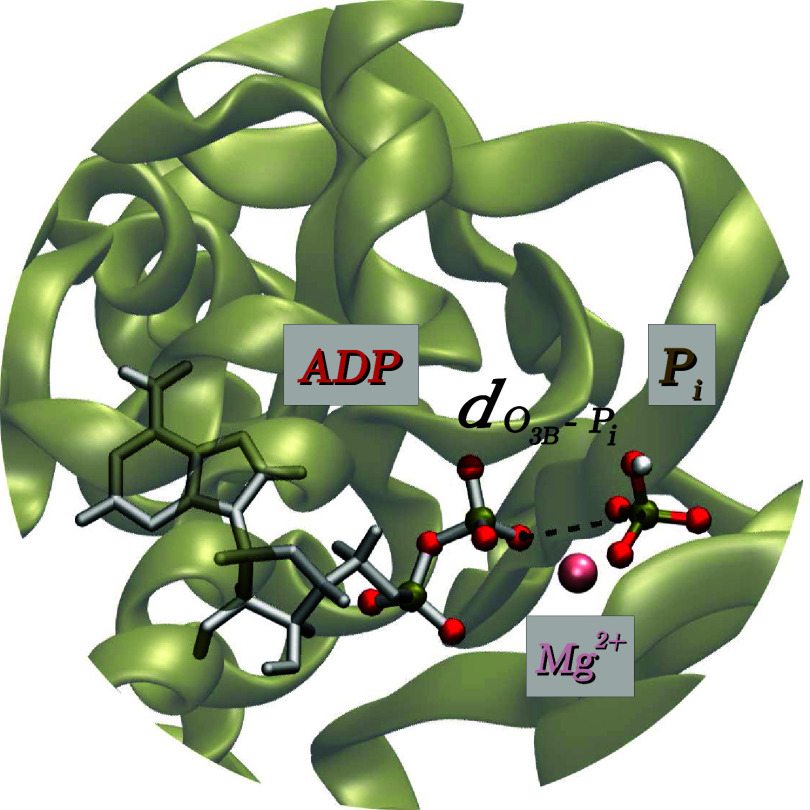
ADP.P_i_ state inside the binding pocket. The pink sphere represents the Mg^2+^ ion that bridges P_i_ to ADP, and the dashed line marks the elongated P_i_ – O_3B_ bond.

The data show that after the cleavage of the P_γ_, the chemical shift of P_β_ undergoes a drastic change and transitions from −16 to −4 ppm (Figure 6). This change is strong enough to change the order of the P_α_ and P_β_ peaks in ATP and ADP, a transformation that would remain undetected without optimizing the molecular structure (see Tables S10 and S11). However, hydrolysis does not have such a significant effect on the P_α_ shift, as it is further spatially removed from the site of the chemical transformation. The chemical shifts of this nucleus fall within the range of −5 to −8 ppm. These two observations are evident both in the experimental findings and in our calculations. After analyzing pre- and posthydrolysis protein trajectories, we calculated chemical shifts starting from an MD trajectory^56^ that simulated a previously hypothesized^23^ intermediate state of the hydrolysis process, ADP.P_i_. The cleaved inorganic phosphate moiety in this case is a singly protonated inorganic phosphate ion: HPO_4_^2–^ (Figure 7).

**Figure 8:**
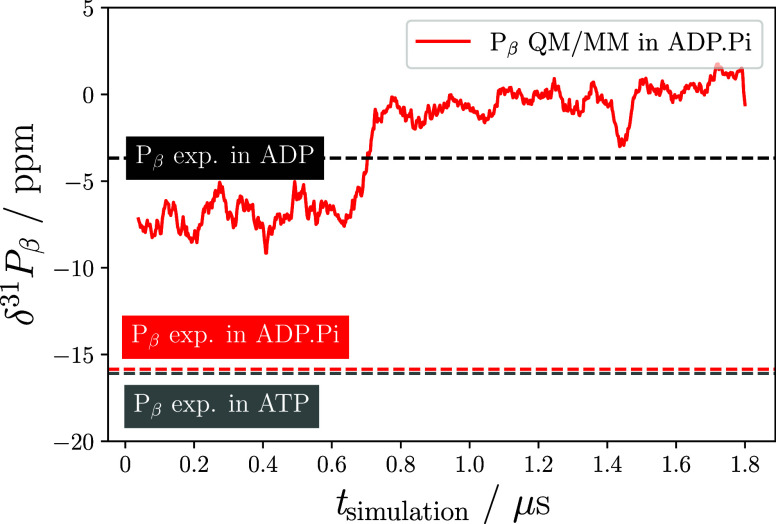
Rolling average of computed P_β_ chemical shifts in the ADP.P_i_ state compared with experimentally measured P_β_ shifts in pre- and posthydrolysis states of p97. For the rolling average, a window size of 20 snapshots was used.

To contextualize our observations, we briefly report findings from QM/MM free energy studies of various enzymatic ATP hydrolysis reactions. These studies provide atomic-level insights into the energetic details of the process and disagree with whether this state is a local energy minimum or rather an unstable transition state structure. Prieß et al.^4^ reported free energy profiles for ATP hydrolysis in an ABC transporter and found two intermediates: ADP + HPO_4_^2–^ (IS1) and ADP + H_2_PO_4_^–^ (IS2). The second intermediate state (IS2) has a high energy, while the first intermediate state (IS1) is similar to ADP.P_i_ in p97, except for the fact that its P_i_–O_3B_ distance falls within the range of 2.75–3.25 Å. Hayashi et al.^13^ found a high-energy quasi-stable intermediate state in F1-ATPase that is separated by very small energy barriers and reported a P_i_ – O_3B_ distance of 2.68 Å. Grigorenko et al.^15^ studied the myosin-catalyzed hydrolysis of ATP and reported no stable intermediates. The reaction product identified on the QM/MM potential energy surface is ADP + HPO_4_^2–^, where the P_i_ – O_3B_ distance corresponds to 2.91 Å. Kiani et al.^12^ found a local energy minimum (ADP.PO_3_^–^) in myosin that is separated by clear energy barriers from the states that precede and follow along the reaction pathway. The P_i_ – O_3B_ distance here is 2.94 Å. They also highlight the importance of a planar metaphosphate (PO_3_^–^) moiety that is a much better target for the nucleophilic attack of the OH^–^ group than the tetrahedral and doubly negative HPO_4_^2–^ found in ADP.P_i_ (Figure 7).

**Table 4:** Deviations in ppm for Predicting ^31^P NMR Chemical Shifts in ATP and ADP Inside the Binding Pocket of p97^a^

			|δ*P*_calc._ – δ*P*_exp._|/ppm			
			P_α_	P_β_	P_γ_	*P̅*
in p97	ATP	MM	0.64	0.40	8.53	3.19
		QM	0.91	3.35	2.41	2.22
	ADP	MM	1.68	9.84	−	5.76
		QM	1.84	0.01	−	0.93

a *P̅* denotes the average deviations.

**Figure 9:**
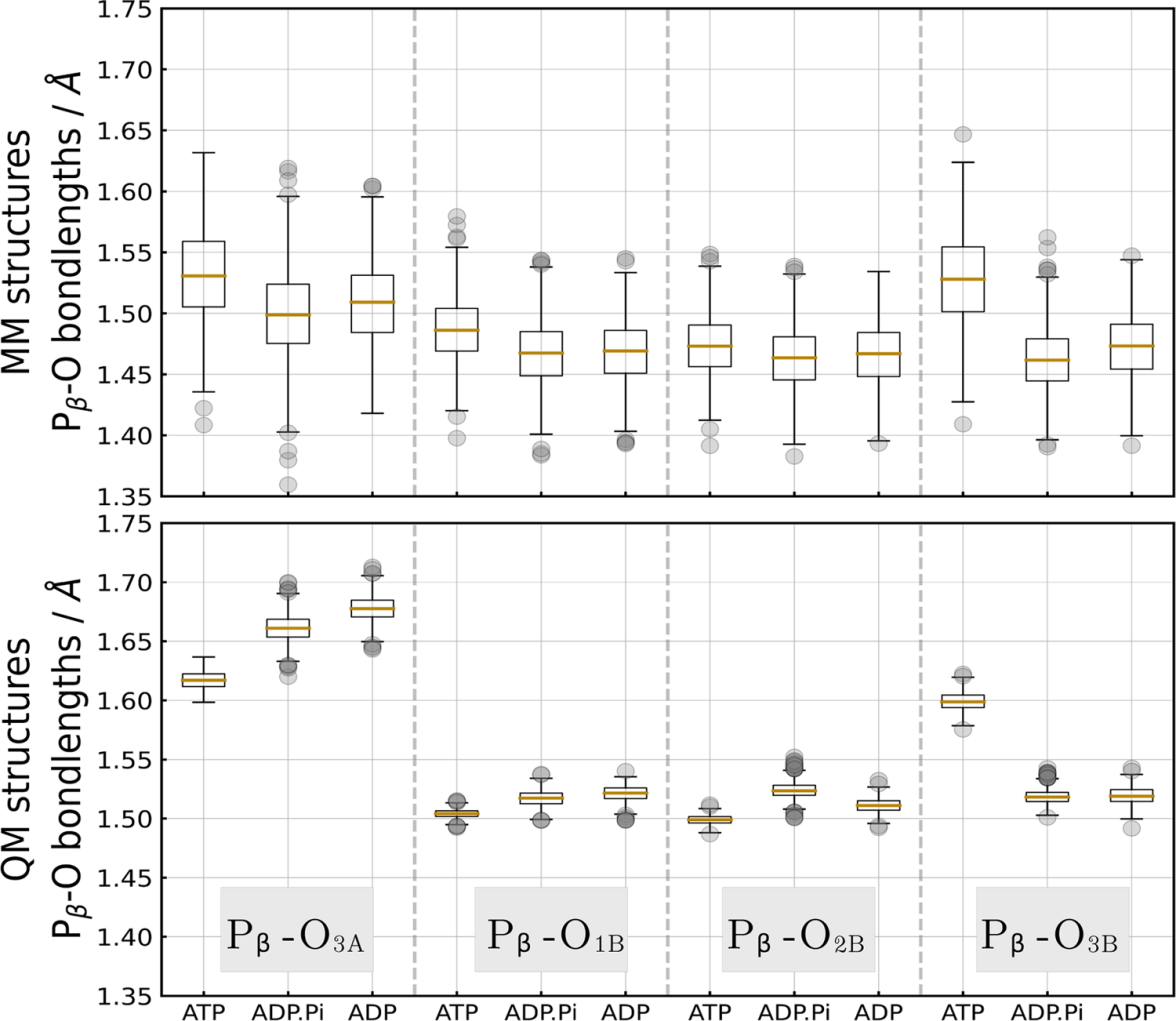
Distribution of the P_β_–O bond lengths in ATP, ADP.P_i_, and ADP molecules bound to p97. The boxes show the interquartile range (IQR), the yellow lines represent the median, whiskers extend to 1.5 times the IQR, and outliers are shown as gray dots.

Throughout the MM-MD trajectory of ADP.P_i_ supplied by Shein et al.,^56^ the P_i_ – O_3B_ distance (see Figure 7) is on average 3.42 Å and remains in the same range after QM/MM structure optimization. In comparison to the intermediate structures in other catalytic environments, this distance appears to be significantly longer. While the P_i_ moiety is indeed trapped inside the binding pocket throughout the 2 μs long simulation, the P_i_ – O_3B_ distance corresponds to a bond that has undergone full cleavage and is notably longer than that of any intermediate found by QM/MM free energy studies. We take this to be the reason we observe large deviations between our predictions and the measured chemical shifts of the ADP.P_i_ intermediate state. In experiment, the observed chemical shift of P_β_ in the ADP.P_i_ state (−15.85 ppm) is very close to the P_β_ shift of ATP (−16.09 ppm, the prehydrolysis state), whereas the computed shifts are much closer to those of P_β_ in ADP (see Figure 8). This is closely linked to the general structural features, the intermediate state closely resembles ADP (see Figure 9 and Section S10 of the SI). Hence, it is not surprising that the computed shifts of ADP.P_i_ are close to the measurements of the posthydrolysis state (Figure 8 and Section S7).

**Figure 10:**
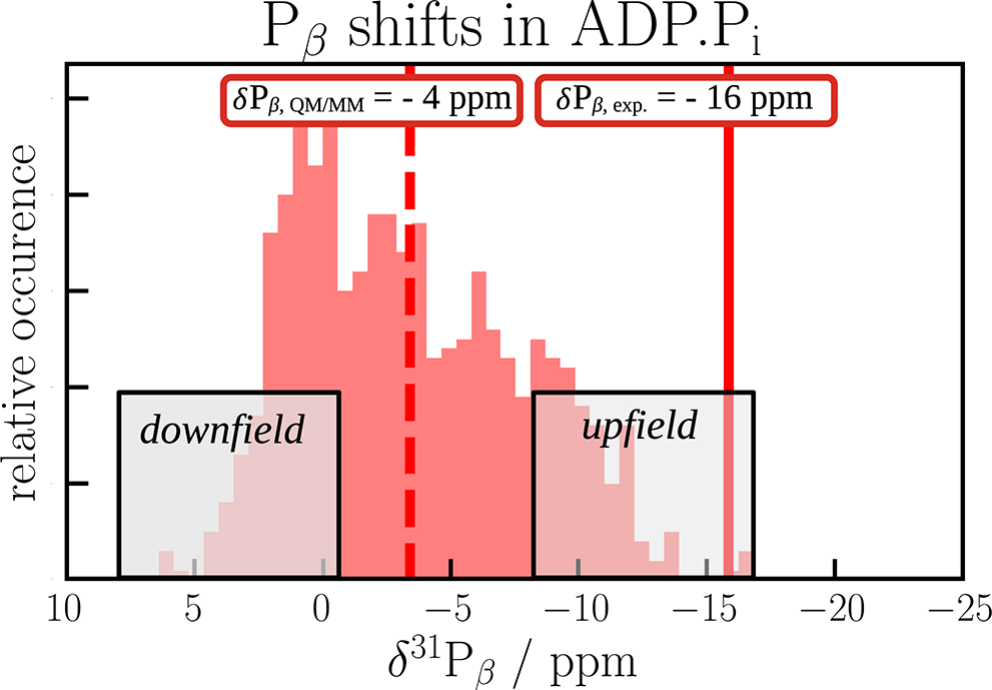
Upfield- and downfield-shifted populations in the distribution of computed P_β_ chemical shifts in ADP.P_i_.

**Figure 11:**
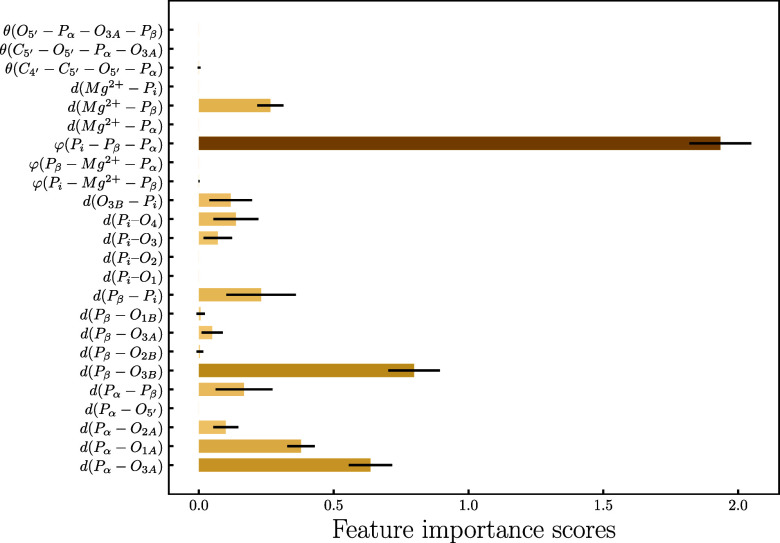
Feature importance scores with error bars predicted from logistic regression.

As we have seen earlier (Figure 6), hydrolysis causes a significant downfield shift of the P_β_ resonances. The distribution of the predicted P_β_ chemical shifts in ADP.P_i_ spans a wide range of 20 ppm (see Figure 10). In order to understand which structural features are dominant in downfield- and upfield-shifted populations and why certain geometries yield shieldings that differ significantly from the experimentally observed ones, a feature importance analysis was carried out (see Figure 11). For this, we selected two populations: 150 MD frames with the most downfield-shifted signals and another 150 frames from the most upfield region (see Figure 10). We measured 24 structural features that represent the chemical environment of the P_β_ nucleus, including interatomic distances, bond angles, and dihedral angles. Notably, these selected features are not all fully independent of each other; see Figure S23 in the SI, where we explored correlations among them. Data analysis based on logistic regression was carried out to assess the significance of these structural features in predicting the outcome of the binary classification problem; for further details about parameters used in the feature importance analysis, see Section S13 of the SI.

Feature importance analysis helped us pinpoint important structural characteristics that strongly impact chemical shieldings. The most important feature we found is the φ(P_i_ – P_β_ – P_α_) angle that depicts the position of P_i_ with respect to ADP and describes how elongated or folded the phosphate backbone is. In contrast to the ATP state of the protein, where the phosphate tail is elongated and the φ(P_i_ – P_β_ – P_α_) angle does not change considerably (see Figure S20), in ADP.P_i_ it changes from 130 to 90° during the MM-MD simulation (see Figure S21). We found that the phosphate backbone is elongated in the first μs of the simulation and later on becomes more folded, a change that can be linked to different side chain conformations of F360 and R359 in the binding pocket.^56^ These two amino acids undergo a correlated motion in the MM-MD trajectory, and as a result, P_i_ and the Mg^2+^ ion change positions. The P_i_ and P_β_ shifts are strongly influenced by these molecular motions, whereas fluctuations in the P_α_ shifts are less marked (see Figure S22). Besides these conformational changes, the phosphate–oxygen bond lengths *d*(P_β_ – O_3B_), *d*(P_α_ – O_3A_), and *d*(P_α_ – O_1A_) proved to be further important features, which in ADP.P_i_ are very similar to those measured in ADP (see Figures 9 and S18).

Evidence from MM-MD simulations, single-particle cryo-electron microscopy (cryo-EM), and NMR measurements indicates that the global protein structure observed for the ADP.P_i_ state of p97 is distinct from states hosting ADP and ATPγS.^56^ However, the findings of our analysis suggest that the structures at the active center sampled by molecular mechanics^56^ represent an ADP with an associated inorganic phosphate in the binding pocket, which does not resemble the intermediate captured by experimental NMR or other intermediates captured by QM/MM studies. We have shown that NMR chemical shifts are very sensitive to subtle changes in the local geometry, and differences in bond lengths as small as 0.10 Å can lead to 10 ppm changes in the ^31^P chemical shifts. Owing to the limited resolution of the X-ray (1.98 Å) and cryo-EM (2.61 Å) structures, many structural details that strongly influence the shieldings remain hidden and thus hinder more accurate chemical shift predictions. An approach to address this problem would entail full QM/MM-MD analysis of the ATP hydrolysis reaction in p97, subsequently followed by QM/MM NMR calculations for the sampled intermediate state, however, due to the extremely high computational cost, this is beyond the scope of the present work.

## 4 Conclusions

The objective of this study was to present a framework for computing ^31^P chemical shifts that combines MM sampling with QM/MM structure optimization and reliable NMR calculations. Our approach was tested in solution, where we showed that simulations can exhibit good agreement with the experiment but only if the sampled phosphate backbone configurations match those observed experimentally. Furthermore, with the presented protocol, we were able to reproduce chemical shifts measured in the pre- and posthydrolysis protein states of p97. However, we compute very different chemical shifts for the postulated ADP.P_i_ intermediate state than observed in the experiment. We cannot safely exclude that the limited resolution of the X-ray structure, from which the dynamic behavior of the protein is explored in molecular mechanics simulations, does not serve as a good starting point for capturing a reaction intermediate by QM/MM NMR calculations.

As our methodology has proven to yield robust results, we infer that the local structures of ADP.P_i_ sampled in the MM-MD are still missing key structural features of the long-lived intermediate measured experimentally. We, therefore, can contribute to this decade-long discussion and conclude that a bonded intermediate has to exist, where the bond is not fully hydrolyzed. A possible future avenue for observing chemical shifts that correspond to a still bonded intermediate structure would be a very costly QM/MM-MD simulation of the phosphate hydrolysis reaction mechanism in p97 followed by NMR calculations.

Complementary to NMR measurements, which provide averaged chemical shifts throughout the acquisition time, our study provides unique insights into how rapid conformational changes in the active site impact chemical shifts. Therefore, we expect our study to serve as a valuable tool in advancing the understanding of predicted chemical shifts and to hold the potential for broader applications involving other NMR active nuclei and biochemical transformations catalyzed by various proteins.

## Supporting Information Available

Details about the MD procedure for nucleotides in solution, experimental details about NMR measurements, settings used for the DFT structure optimization and NMR calculations, details about the effect of the structure optimization on molecular structures, the QM/MM setup for calculations inside the protein, a QM region size convergence study, the exact QM region sizes used for the NMR calculations, tables with the used internal references, predicted chemical shifts in solution and in protein environment, parameters used in the feature importance analysis, and a heatmap exploring correlations among structural features (PDF)
