# Supplementary material for: Systematic QM/MM Study for Predicting 31P NMR Chemical Shifts of Adenosine Nucleotides in Solution and Stages of ATP Hydrolysis in a Protein Environment
Source: J Chem Theory Comput. 2024 Mar 18;20(6):2433–44. doi: 10.1021/acs.jctc.3c01280 (PMC10976643; doi:10.1021/acs.jctc.3c01280)
Supplement: Supplementary file 1 — ct3c01280_si_001.pdf [file ct3c01280_si_001.pdf]

Supplementary Material: A systematic QM/MM study for predicting ^{31}P NMR chemical shifts of adenosine nucleotides in solution and stages of ATP hydrolysis in a protein environment

Judit Katalin Szántó,[1] Johannes C. B. Dietschreit,[1,2]
Mikhail Shein,[3] Anne K. Schütz,[3] Christian Ochsenfeld*,[1,4]

[1]Chair of Theoretical Chemistry, Department of Chemistry,
University of Munich (LMU), Butenandtstr. 7, D-81377 München, Germany,
[2]Department of Materials Science and Engineering, Massachusetts Institute of Technology,
Cambridge, Massachusetts 02139, USA
[3]Department of Chemistry, University of Munich (LMU),
Butenandtstr. 5-13, D-81377 München, Germany
[4]Max Planck Institute for Solid State Research,
Heisenbergstr. 1, D-70569 Stuttgart, Germany
*E-Mail: christian.ochsenfeld@uni-muenchen.de

Contents

1 MD procedure for nucleotides in solution S3

2 NMR measurements for nucleotides in solution S4

3 Settings used for DFT structure optimization calculations S5

4 Settings used for DFT NMR calculations in solution and inside the protein S6

5 Predicted chemical shifts for nucleotides in solution S6

6 QM/MM setup for calculations inside p97: Defining the QM region S9

7 Predicted chemical shifts for nucleotides in protein environment, bound to p97 S10

8 Size convergence study S13

9 QM region sizes used for the NMR calculations in solution and in protein environment S19

10 Changes in the P_{α}-O bond lengths of the ATP, ADP.P_{i}, and ADP molecules in p97 before and after structure optimization S19

11 Changes in the $P_{\alpha} - P_{\beta} - P_{\gamma}$ angle of solvated ATP S21

12 Changes in the $P_{\alpha} - P_{\beta} - P_{\gamma}$ angle of ATP and ADP.P_{i} inside the binding pocket of p97 S22

13 Feature importance analysis with logistic regression S23

14 Correlation analysis for the selected structural features in ADP.P_{i} S24

References S25

1 MD procedure for nucleotides in solution

Before carrying out calculations in protein environment we validated our methodology for solvated ATP and ADP molecules. To study how ionic concentrations affect chemical shifts, we performed MD simulations using different ionic compositions. The MD simulations were carried out with explicit solvent. The cuboid simulation boxes were populated with TIP3P water molecules. Molecular dynamics (MD) simulations were performed with the NAMD 2.10 program package[1]. ADP and ATP structures and parameters were taken from the parameter database of the University of Manchester [2]. The nucleotides were solvated in a cuboid box of TIP3P water [3], the edge size was chosen such that water/nucleotide ratio was close to that in experiment.

For the minimal ionic concentration simulations (min. ionic cond.), the smallest possible number of Mg^{2+} and Cl^- counterions was used to neutralize the negative charges of the ADP and ATP molecules. The minimal ionic concentrations used in the experiment were: 5 mM nucleotide, 10 mM $MgCl_2$. In contrast, the high ionic concentration simulations (high ionic cond.) contain more ions and thus mimic the ionic strength of the high salt buffer used in the experiments: 5 mM nucleotide, 25 mM NaCl, 10 mM $MgCl_2$, 50 mM KCl. The charge neutrality of the simulation box is ensured in all MD simulations.

Table S1: Number of counter ions and water molecules used in the MD simulations of nucleotides in solution. The parameter l is the side length of the simulation box.

		Na^+	Mg^{2+}	K^+	Cl^-	solvent molecules	l / Å
**min. ionic cond.**	ADP	-	2	-	1	800	40
	ATP	-	2	-	-	950	40
**high ionic cond.**	single ADP	5	1	5	9	5600	60
	two ADP	11	1	10	17	11000	75
	single ATP	6	1	5	9	5600	60
	two ATP	13	1	10	17	11000	75

Hydrogen vibrations were suppressed with the SHAKE algorithm [4]. Periodic electrostatic interactions were evaluated with the Particle Mesh Ewald method and van der Waals interactions were truncated at a cutoff distance of 12 Å. The integration time step was 2 fs. In production and equilibration, we performed simulations in the NPT ensemble, we used Langevin dynamics for temperature control and Langevin piston Nosé-Hoover method for pressure control. The friction constant was set to 1 amu/ps, the target pressure to 1.01325 bar, the piston oscillation period to 200 fs, the piston damping time scale to 50 fs and the barostat noise temperature was set equal to the target temperature, 300K. The simulation boxes were heated over 30 ps from 0 to 300 K, followed by an equilibration run of 100 ps. Starting from the equilibrated structures we performed a single production run of 100 ns for each system.

2 NMR measurements for nucleotides in solution

Sample preparation

Experimental ^{31}P chemical shifts of ATP and ADP in solution were determined in two different buffer systems, referred to as minimal and high ionic conditions, whose compositions are given in Table S2. Briefly, ADP (Sigma-Aldrich, USA) or ATP (SERVA, Germany) were dissolved into the respective buffers to a final concentration of 5 mM and a volume of 500 µL and placed in a 5 mm NMR tube.

Table S2: Composition of buffers used for determination of experimental nucleotide shifts.

Buffer identity	Composition
Minimal ionic cond.	5 mM nucleotide, 10 mM HEPES, 10 mM $MgCl_2$, 1 mM DSS, 5% D_2O, pH 7.5
High ionic cond.	5 mM nucleotide, 10 mM HEPES, 10 mM $MgCl_2$, 1 mM DSS, 5% D_2O, 25 mM NaCl, 50 mM KCl, pH 7.5

NMR measurements

Solution-state NMR spectra were recorded on a Bruker 400 MHz spectrometer equipped with a room temperature BBO probe. Sample temperature was set to 298 K. A directly pulsed 31P experiment was used. The sweep width was set to 100 ppm, the transmitter frequency offset to -5 ppm, the inter-scan delay to 5 s, the acquisition time to 125 ms, the number of scans to 128. Spectra were processed by applying an exponential window function with a line broadening of 5 Hz and by zero-filling to four times the original size. All spectra were calibrated to an internal reference (DSS). Peaks were assigned based on reported ^{31}P NMR chemical shifts [5].

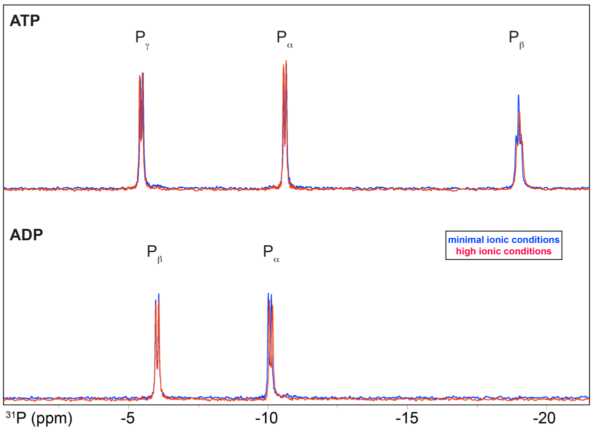

Figure S1: ^{31}P NMR spectra of ATP and ADP measured in minimal and high ionic conditions. Directly pulsed ^{31}P 1D NMR spectra of ATP (top) and ADP (bottom) were acquired under minimal (blue) and high (red) ionic conditions at 298 K.

3 Settings used for DFT structure optimization calculations

In order to improve the configurations sampled by MM-MD we optimized the structure of the nucleotides prior to the NMR computations. The DL-Find library [6] implemented in PyChemShell [7] was used for structure optimizations in the protein environment. For the solvated nucleotides we used geomeTRIC [8], connected to the Python-interface of FermiONs++[9–11]. Convergence criteria for DFT QM/MM structure optimizations were set as follows:

Table S3: Left: Convergence criteria for structure optimization of solvated ADP and ATP. Right: Convergence criteria for structure optimization of ADP, ADP.P_i and ATP in p97

Criteria	Threshold
Energy	1×10^{-4} E_h
RMS gradient	5×10^{-3} E_h
Max. gradient	1×10^{-2} E_h
RMS step	5×10^{-3} E_h
Max. step	1×10^{-2} E_h

Criteria	Threshold
Energy	4×10^{-4} E_h
RMS gradient	1×10^{-3} E_h
Max. gradient	8×10^{-3} E_h
RMS step	5×10^{-3} E_h
Max. step	5×10^{-1} E_h

4 Settings used for DFT NMR calculations in solution and inside the protein

We carried out NMR calculations with the FERMIONS++ program package[9–13]. Both for calculations in solution and in protein environment the same settings and integral thresholds were used. The SCF convergence criterion was defined as the root mean square (RMS) of the FP commutator and set to 10^{-7} a.u. In the SCF algorithm the Loewdin orthogonalization method was used to orthogonalize the atomic orbitals. As initial guess for the SCF density we used the superposition of atomic densities ('ascf') which were generated on-the fly. To accelerate the SCF convergence Pulay's DIIS scheme was used with the DIIS start criterion and subspace dimension set to 10. The start criterion determines when the DIIS method is initiated during the iterative process and the subspace dimension refers to the number of past electronic states considered in the DIIS calculation.

We applied the seminumerical evaluation of the exchange parts (sn-LinK). We used the g1 integration grid for DFT and sn-LinK. The general integral threshold was 10^{-12}, the pre-selection threshold for PreLinK was set to 10^{-6}. To determine significant shell-pairs we used 10^{-14} as threshold and 10^{-15} as significance threshold for single primitive shell-pairs.

With increasing system size calculations in the protein environment showed a poor convergence behaviour, due to smaller HOMO-LUMO gaps. In order to avoid convergence problems and ensure the correctness of the computed chemical shifts, combined with DIIS, a dynamic damping algorithm was applied using the Saunders-Hillier scheme. As the orthonormality of the MO-vectors can deteriorate after a number of SCF cycles, Gram-Schmidt orthogonalization was performed after every 5th cycle.

We employed the same QM size of 3.8 Å around the nuclei of interest for the QM/MM NMR calculations and the structure optimizations. For further details about the QM region sizes see Table S13 and Table S14.

5 Predicted chemical shifts for nucleotides in solution

In QM NMR calculations, the primary results are absolute magnetic shieldings, which represent the change in the magnetic environment experienced by a nucleus due to its local electron density and the external magnetic field. The choice of the referencing method is critical for the interpretation of the computed chemical shifts. In our internal referencing scheme, the P_α nucleus of the nucleotides was selected, because it is not directly involved in the hydrolysis and its immediate chemical environment does not change strongly between ATP and ADP. When computing chemical shifts, simulations carried out under minimal ionic conditions were referenced separately from high ionic condition simulations and solvated ADP and ATP simulated under the same conditions were referenced identically. We also treated geometries obtained directly from the molecular mechanics (MM) simulations differently from the geometry-optimized structures, as shown in Table S4 and Table S5. This approach allows us to consider and differenti-

ate the various effects introduced by both increased ionic concentration and structure optimization.

Table S4: Internal reference used for isotropic shieldings predicted from MM structures of ADP and ATP in solution. The reference nucleus is P_α.

			$\sigma_{calc.}$	$\delta_{exp.}$ /ppm	$\sigma_{calc.} + \delta_{exp.}$ /ppm	$ref = \overline{\sigma_{\text{calc.}} + \delta_{\text{exp.}}}$
min. ionic cond.	ATP		337.03	-10.62	326.41	329.07 ppm
	ADP		341.80	-10.07	331.73	
high ionic cond.	single ATP		338.74	-10.61	328.13	328.05 ppm
	single ADP		339.86	-10.12	329.74	
	two ATP	ATP1	337.96	-10.61	327.35	
		ATP2	337.00	-10.61	326.39	
	two ADP	ADP1	337.12	-10.12	327.00	
		ADP2	339.8	-10.12	329.68	

Table S5: Internal reference used for isotropic shieldings predicted from QM/MM optimized structures of ADP and ATP in solution. The reference nucleus is P_α.

			$\sigma_{calc.}$	$\delta_{exp.}$ /ppm	$\sigma_{calc.} + \delta_{exp.}$ /ppm	$ref = \overline{\sigma_{\text{calc.}} + \delta_{\text{exp.}}}$
min. ionic cond.	ATP		323.53	-10.62	312.91	314.67 ppm
	ADP		326.49	-10.07	316.42	
high ionic cond.	single ATP		319.78	-10.61	309.17	309.19 ppm
	single ADP		318.13	-10.12	308.01	
	two ATP	ATP1	320.10	-10.61	309.49	
		ATP2	319.82	-10.61	309.21	
	two ADP	ADP1	319.89	-10.12	309.77	
		ADP2	319.60	-10.12	309.48	

Table S6: Calculated and measured ^{31}P NMR chemical shifts for the ATP molecule in solution

				δ ^{31}P / ppm		
				P_α	P_β	P_γ
min. ionic cond.	*calc.*		MM	-7.96	-23.19	-18.74
			QM	-8.86	-21.30	-7.42
	measured			-10.62	-19.00	-5.47
high ionic cond.	*calc.*	single ATP	MM	-6.70	-13.66	-14.00
			QM	-10.58	-13.93	-2.56
		two ATP	MM	-8.47	-20.10	-13.90
			QM	-10.60	-18.01	-3.85
	measured			-10.61	-19.04	-5.45

Table S7: Calculated and measured ^{31}P NMR chemical shifts for the ADP molecule in solution

				δ ^{31}P / ppm	
				P_α	P_β
min. ionic cond.	*calc.*		MM	-12.73	-19.11
			QM	-11.82	-8.28
	measured			-10.07	-6.03
high ionic cond.	*calc.*	single ADP	MM	-8.74	-14.24
			QM	-8.94	-4.39
		two ADP	MM	-8.21	-11.22
			QM	-10.51	-3.75
	measured			-10.12	-6.03

6 QM/MM setup for calculations inside p97: Defining the QM region

When defining the QM region used in structure optimization and NMR calculations, we opted to cut only non-polar C-C bonds. In this way we avoided breaking peptide bonds and cutting through the polar C-N bonds, and thus circumvented unintended polarization effects at the QM/MM boundary. An automatic workflow was employed to place H-atoms as links between the MM and QM region. Link atoms were introduced between C_β and C_α atoms if single amino acid were selected into the QM region (Figure S2 a) and between C_α and C atoms in case of an amino acid sequence (Figure S2 b). The charge for all snapshots extracted from the trajectory was calculated based on the charge of the central nucleotide (ATP/ADP.P_i/ADP), selected protein residues, and surrounding ions.

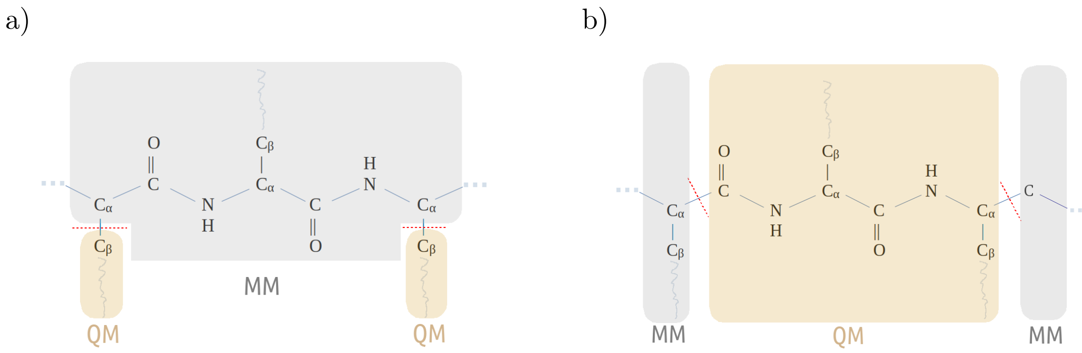

Figure S2: Representation of QM/MM partitioning scheme: a) cutting through the C_α and C_β bond when including single amino acids in the QM region and b) cutting along the protein backbone between C_α and C atoms when including a series of amino acids.

7 Predicted chemical shifts for nucleotides in protein environment, bound to p97

Similar to calculations in solution, we chose the P_α nucleus for our internal reference and treated shifts predicted from MM and QM optimized structures separately.

Table S8: Internal reference used for isotropic shieldings predicted from MM structures of ATP, ADP.P_i, and ADP in p97. The reference nucleus is P_α.

	$\sigma_{calc.}$/ppm	$\delta_{exp.}$/ppm	$(\sigma_{calc.} + \delta_{exp.})$/ppm	$ref = \overline{\sigma_{\text{calc.}} + \delta_{\text{exp.}}}$
P_α in ATP	337.71	-5.80	331.91	
P_α in ADP.P_i	339.88	-5.00	334.88	332.55 ppm
P_α in ADP	338.76	-7.89	330.87	

Table S9: Internal reference used for isotropic shieldings predicted from QM/MM optimized structures of ATP, ADP.P_i, and ADP in p97. The reference nucleus is P_α.

	$\sigma_{calc.}$/ppm	$\delta_{exp.}$/ppm	$(\sigma_{calc.} + \delta_{exp.})$/ppm	$ref = \overline{\sigma_{\text{calc.}} + \delta_{\text{exp.}}}$
P_α in ATP	323.33	-5.80	317.53	
P_α in ADP.P_i	322.54	-5.00	317.54	316.62 ppm
P_α in ADP	322.67	-7.89	314.78	

Table S10: Calculated and measured ^{31}P NMR chemical shifts for the ADP molecule inside the binding pocket of p97

		δ ^{31}P / ppm	
		P_α	P_β
calc.	MM	-6.21	-13.51
	QM	-6.05	-3.68
measured		-7.89	-3.67

Table S11: Calculated and measured ^{31}P NMR chemical shifts for the ATP molecule inside the binding pocket of p97

		δ ^{31}P / ppm		
		P_α	P_β	P_γ
calc.	MM	-5.16	-16.49	-17.19
	QM	-6.71	-12.74	-6.25
measured		-5.80	-16.09	-8.66

Table S12: Calculated and measured ^{31}P NMR chemical shifts for ADP.P_i inside the binding pocket of p97.

		δ ^{31}P / ppm		
		P_α	P_β	P_i
calc.	MM	-7.33	-14.78	2.61
	QM	-6.06	-2.95	5.87
measured		-5.00	-15.85	multiple peaks: -2.1, -2.9, -6.8, -9.2, -10.9, -12.0

At first sight, the chemical shift of P_β predicted in ADP.P_i without structure optimization (MM: -14.78 ppm) (Table S12) might seem to agree with experiment (measured: -15.85 ppm), but in fact, they seem to be agreeing for the wrong reason. Both before (MM: -14.78 ppm) and after structure optimization (QM: -2.95 ppm) the P_β chemical shift of ADP.P_i are very similar to the post-hydrolysis P_β shifts (MM: -13.51 ppm, QM: -3.68 ppm) specific to the ADP molecule. In contrast, the experimentally measured chemical shifts of ADP.P_i (measured: -15.85 ppm) are very similar to pre-hydrolysis chemical shifts (measured: -16.09 ppm). The QM prediction for the P_β in ADP.P_i is far in the downfield region. We observe a similar effect in the same direction for the P_β in ATP, with smaller errors compared to the experimental data.

The time evolution curves show that in contrast to P_β (Figure 8 in the main manuscript) and P_i nuclei (Figure S4), chemical shifts predicted for the P_α nucleus (Figure S3) are less affected by environmental effects and structural changes in the active site.

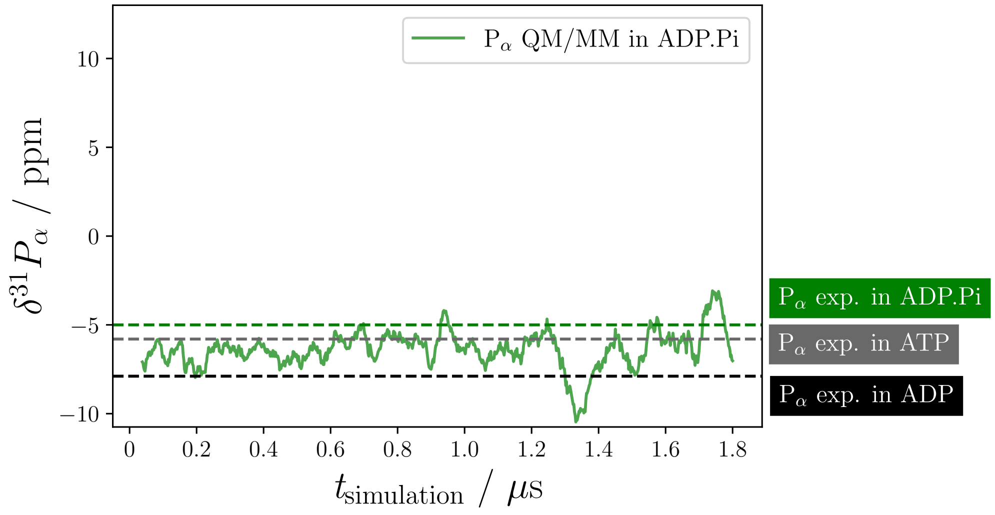

Figure S3: Computed P_α chemical shift (continuous line) in the ADP.P_i state compared to experimentally measured P_α shifts (dashed lines) in p97. For the rolling average a window size of 20 snapshots was used.

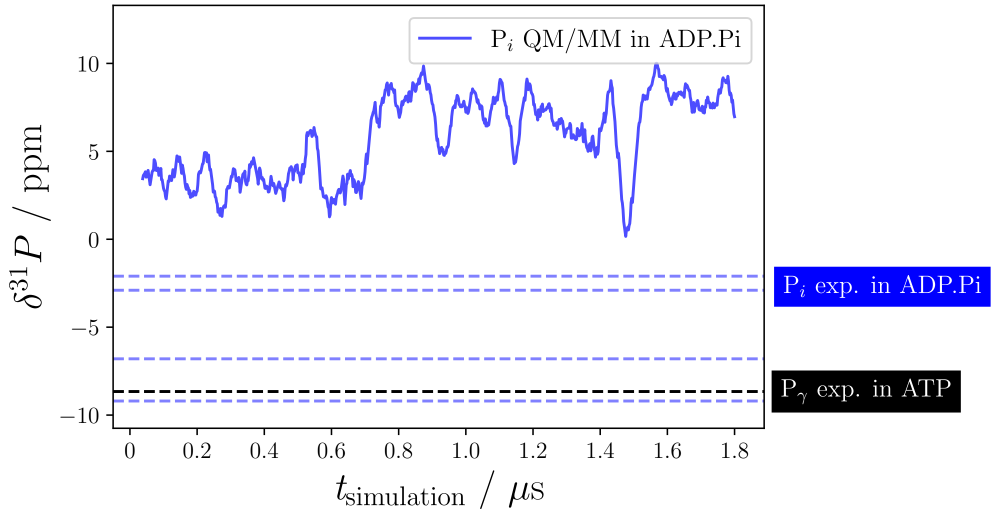

Figure S4: Computed P_i chemical shifts (continuous line) in the ADP.P_i state compared to experimentally measured P_i peaks (blue dashed lines) and P_γ shifts (black dashed line) in p97. For the rolling average a window size of 20 snapshots was used.

8 Size convergence study

We performed a benchmark study to investigate long-range effects - such as electrostatic interactions with charged amino acids/ions, the influence of various side chain conformations of amino acids - and the overall size convergence behavior of the investigated systems. QM/MM NMR calculations were carried out at the B97-2/pcSseg-2 [14, 15] level of theory. The QM region was systematically increased, including more neighboring solvent molecules, ions, and amino acids until changes in the NMR shieldings between subsequent QM-sphere increases are negligible (0.5 ppm) for the considered ^{31}P nuclei. The QM spheres were defined by a distance criterion ranging from 2 Å to 4 Å in steps of 0.5 Å, i.e., a molecule is assigned to the QM part, if any atom of the molecule is within the cutoff distance to any atoms of the central region of interest. The QM region for simulations in solution is selected around the ADP and ATP molecules. In protein environment, for the sake of computational efficiency we excluded the adenine ring and increased the QM region by selecting residues of the protein environment around the ribose ring and the phosphate backbone of ADP, ADP.P_i, and ATP, respectively.

The figures below illustrate the approach to studying the convergence behavior with increasing the size of the QM region for simulations in solution (single nucleotides, min. ionic cond. - Figure S5) and in protein environment (Figure S10).

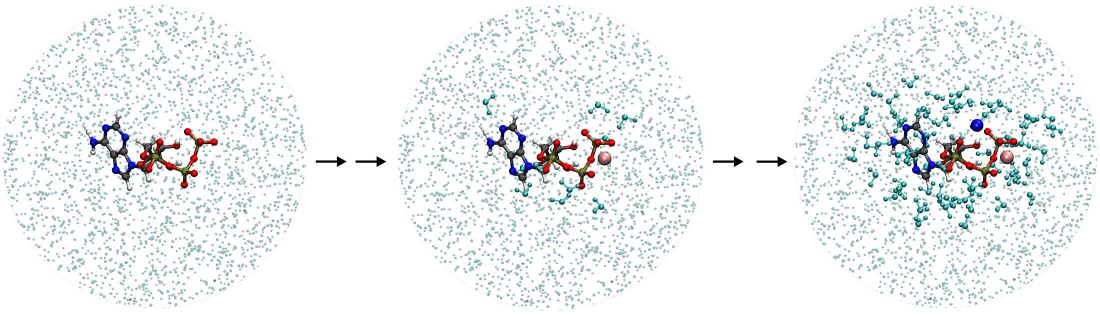

Figure S5: Illustration of the increasing QM regions used in the size convergence study for the QM/MM calculations carried out in solution

For testing the size convergence, 5 equally spaced frames were selected from MD trajectories and 10 different QM regions were used.

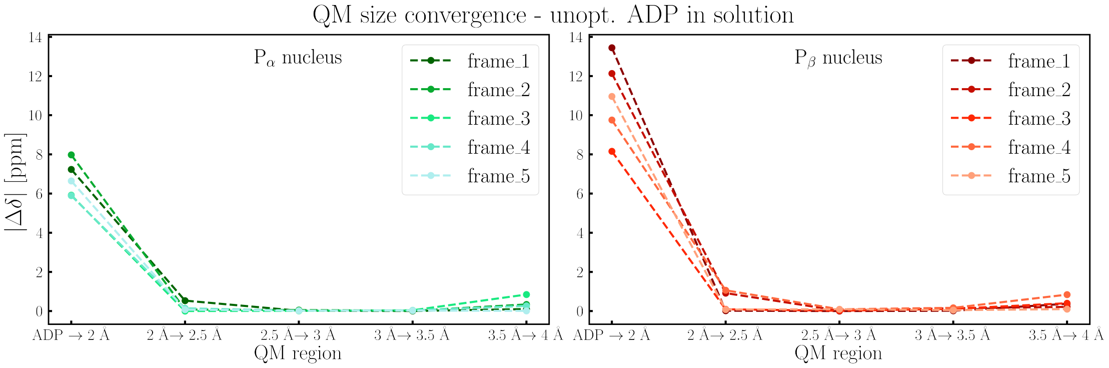

Figure S6: Demonstration of the QM size convergence for the ADP molecule in solution (MM geometries)

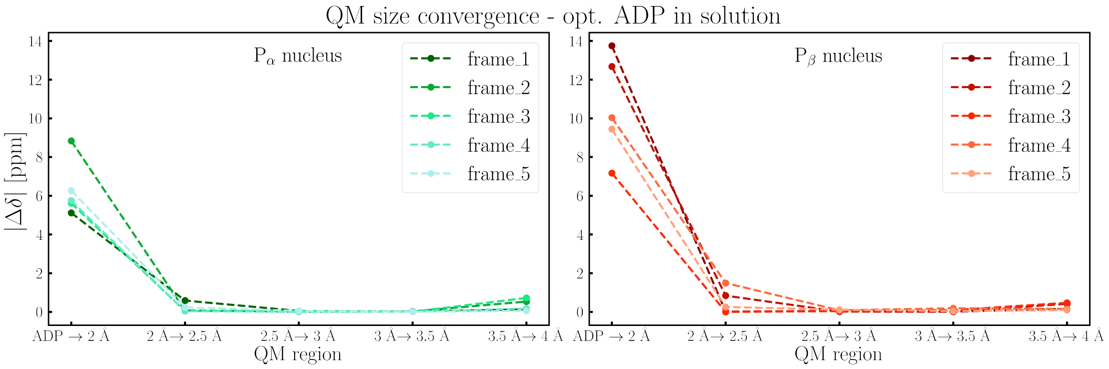

Figure S7: Demonstration of the QM size convergence for the ADP molecule in solution (QM opt. geometries)

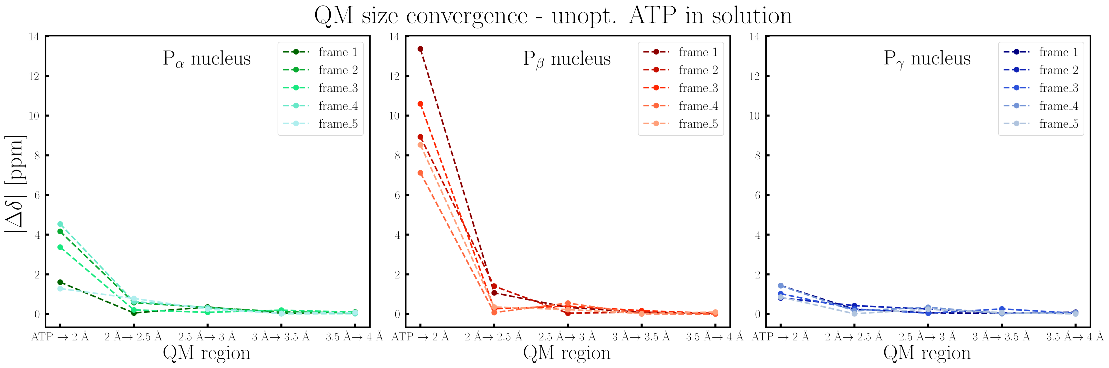

Figure S8: Demonstration of the QM size convergence for the ATP molecule (MM geometries) in solution

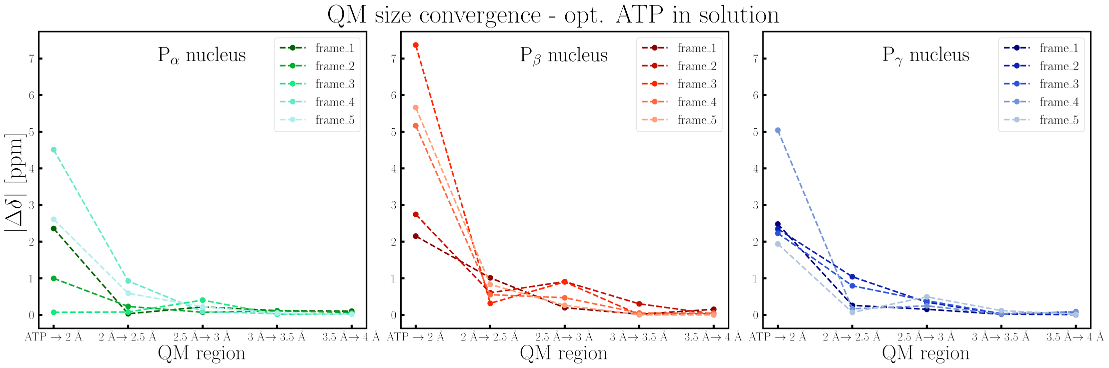

Figure S9: Demonstration of the QM size convergence for the ADP molecule (QM opt. geometries) in solution

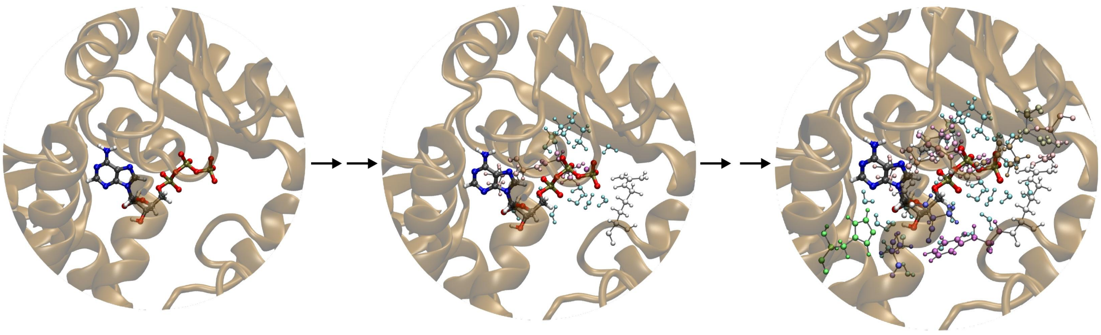

Figure S10: Illustration of the increasing QM regions used in the size convergence study for the QM/MM calculations carried out with p97

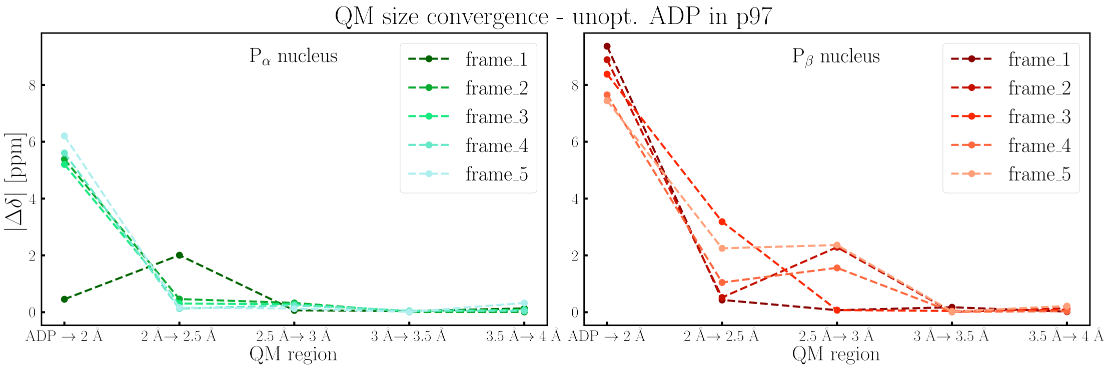

Figure S11: Demonstration of the QM size convergence for the ADP molecule (MM geometries) inside the binding pocket

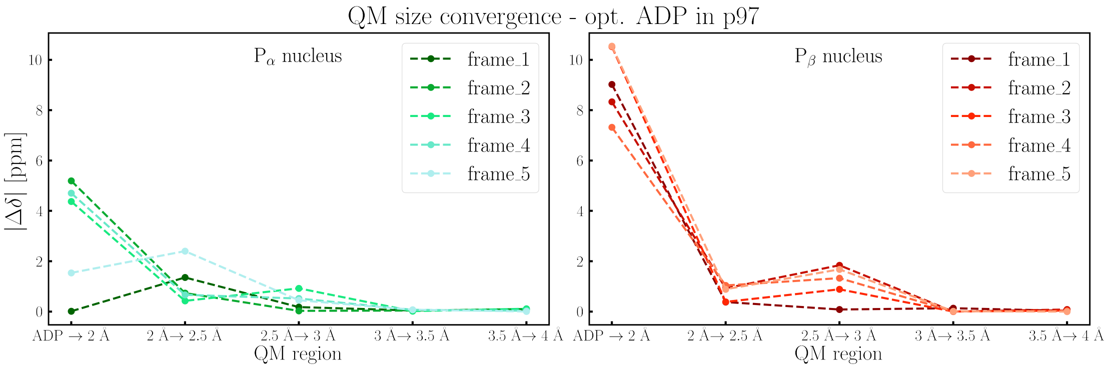

Figure S12: Demonstration of the QM size convergence for the ADP molecule (QM opt. geometries) inside the binding pocket

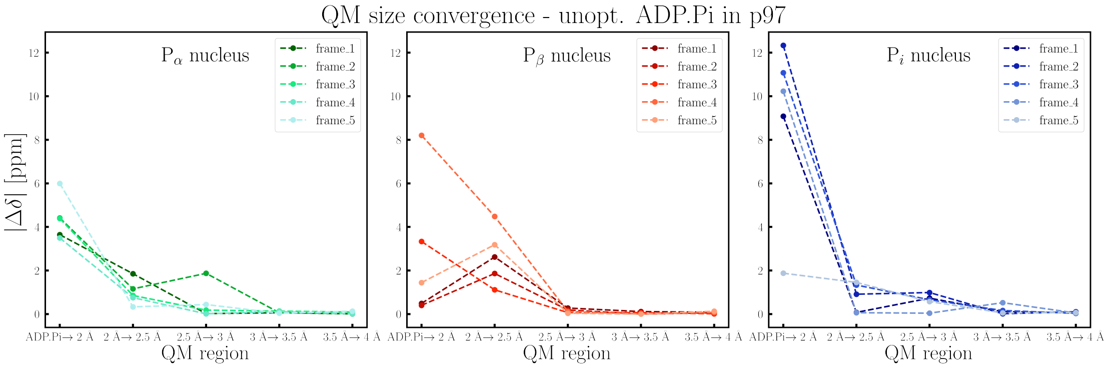

Figure S13: Demonstration of the QM size convergence for the ADP.P_i (MM geometries) intermediate state inside the binding pocket

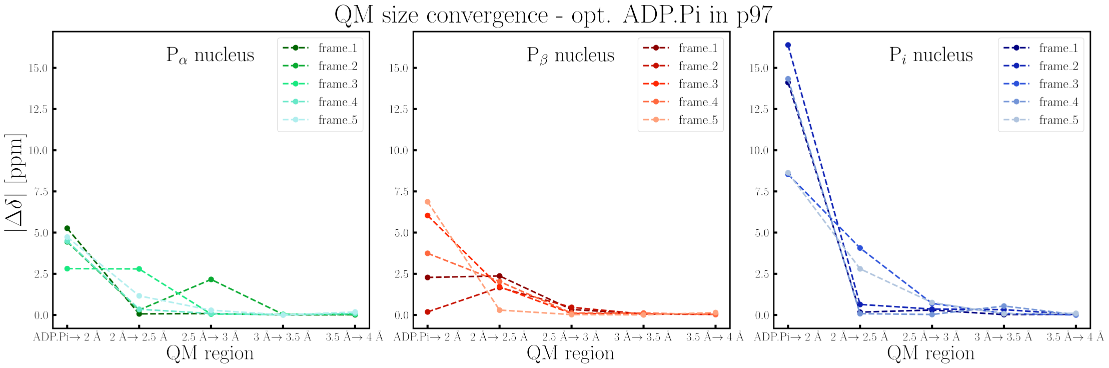

Figure S14: Demonstration of the QM size convergence for the ADP.P_i (QM opt. geometries) intermediate state inside the binding pocket

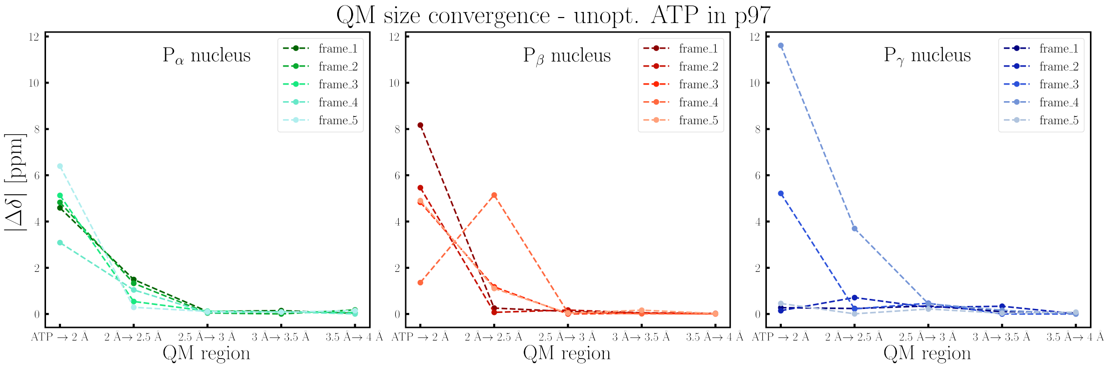

Figure S15: Demonstration of the QM size convergence for the ATP molecule (MM geometries) inside the binding pocket

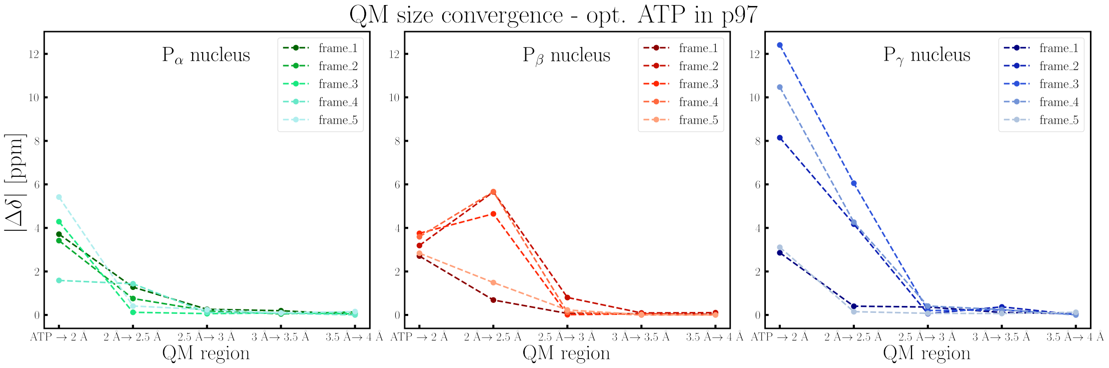

Figure S16: Demonstration of the QM size convergence for the ATP molecule (QM opt. geometries) inside the binding pocket

We can conclude that size convergence is reached when 4.0 Å of the environment is treated at the QM level. Already, increasing the QM sphere from 3.5 to 4.0 Å does not lead to averaged changes larger than 0.3 ppm in the computed P_α, P_β and P_γ isotropic shieldings in the optimized structures.

9 QM region sizes used for the NMR calculations in solution and in protein environment

The actual number of atoms in the QM region varies from frame to frame due to small changes in the structure of the nucleotides. The following tables contain the average numbers of QM atoms used in the NMR calculations of different trajectories in solution (Table S13) and in protein environment (Table S14).

Table S13: Average number of QM atoms used in the NMR calculations of nucleotides in solution

	**min. ionic cond.**		**high ionic cond.**			
	ADP	ATP	single ADP	two ADP	single ATP	two ATP
QM atoms	213	220	209	226	229	251

Table S14: Average number of QM atoms used in the NMR calculations of nucleotides bound to p97

	**in p97 ATPase**		
	ATP	ADP.P_i	ADP
QM atoms	464	719	490

10 Changes in the P_α-O bond lengths of the ATP, ADP.P_i, and ADP molecules in p97 before and after structure optimization

The P-O bond lengths of the hypothesized ADP.P_i reaction intermediate are compared to pre- and post-hydrolysis states. A uniform distribution of the P-O bonds is observed in the force field ensembles (MM structures), while after the structure optimization, an overall lengthening of all P-O bonds can be seen.

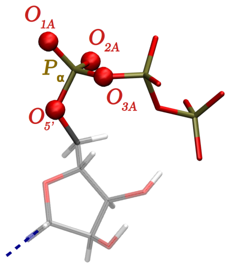

Figure S17: P_α-O bonds of the phosphate backbone in ATP

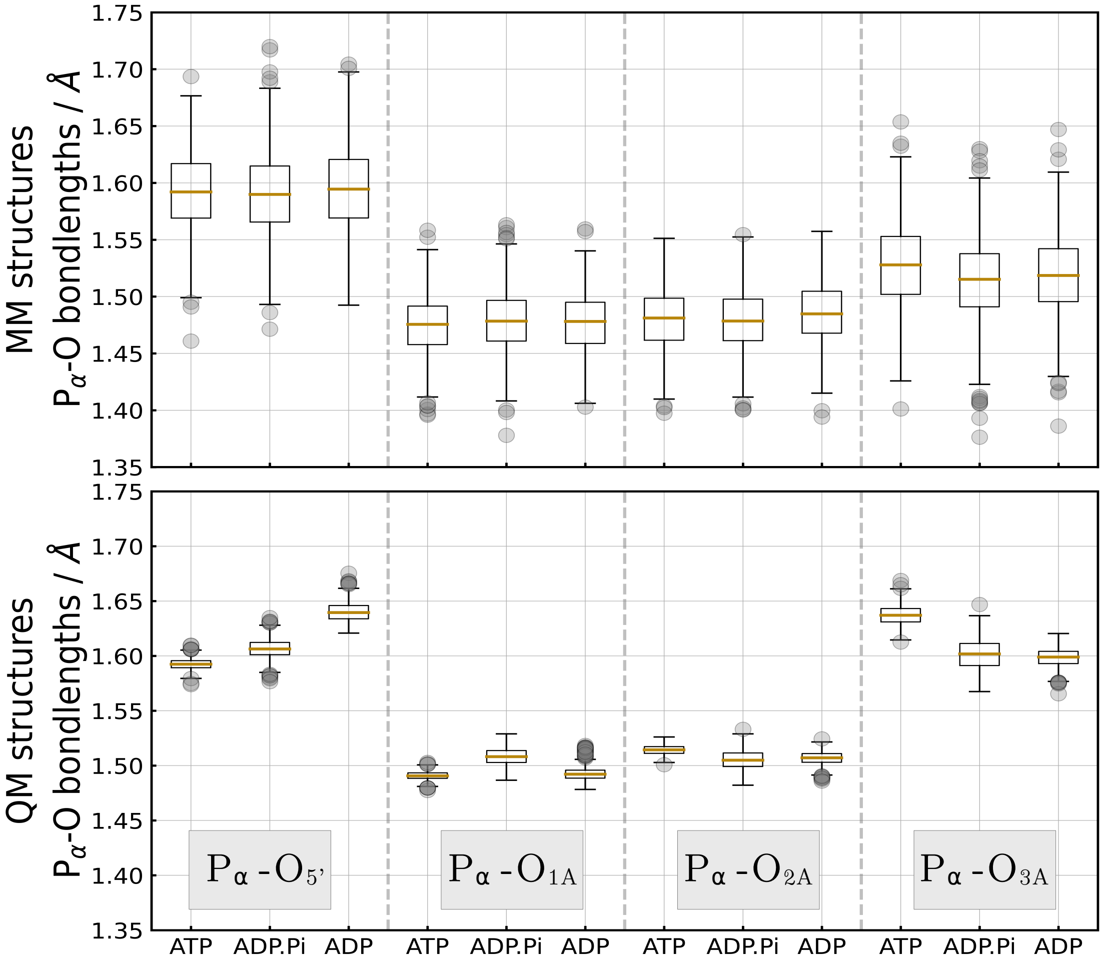

Figure S18: The distribution of the P_α-O bond lengths in ATP, ADP.P_i, and ADP molecules bound to p97. The boxes show the interquartile range (IQR), the yellow lines represent the median, whiskers extend to 1.5 times the IQR and outliers are shown as gray dots.

11 Changes in the $P_\alpha - P_\beta - P_\gamma$ angle of solvated ATP

The inspection of the $P_\alpha - P_\beta - P_\gamma$ angle reveals two different ATP conformers in solution. One conformer is characterized by an elongated phosphate tail (high ionic cond. - single ATP), whereas the other conformer exhibits a more folded phosphate backbone (min. ionic cond. ATP, high ionic cond. - two ATP). In force field ensembles these two conformers predominate, distinguished by bidentate (elongated phosphate tail) and tridentate (folded phosphate tail) Mg^{2+}-phosphate coordination [16].

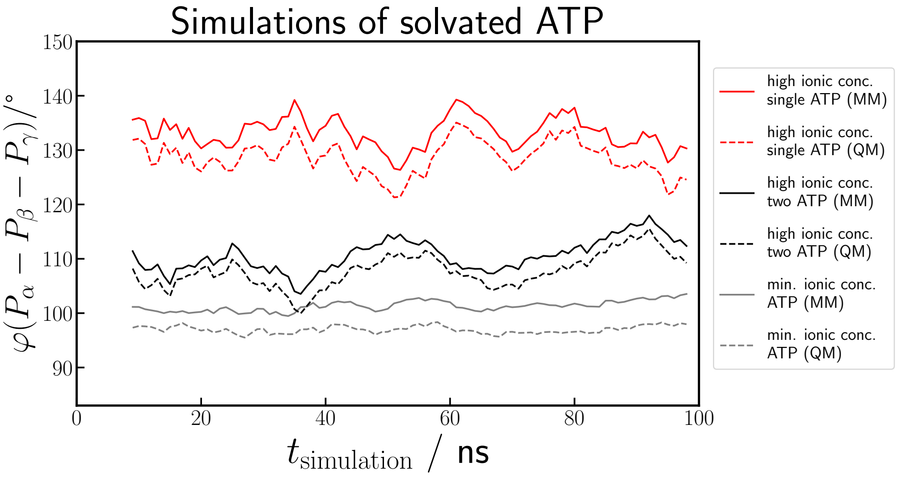

Figure S19: Time evolution of $P_\alpha - P_\beta - P_\gamma$ angles in solvated ATP. For the rolling averages a window size of 10 frames was used.

This structural difference has an impact on the ^{31}P NMR chemical shifts. We see larger errors compared to experimental values when we predict chemical shifts from the trajectory that samples the conformer with the more extended phosphate tail (see Table 3, high ionic conc. single ATP - main manuscript). With QM/MM optimization these errors are reduced, we find the closest local minimum by slightly changing the bond angles and bond lengths but transitioning from one local minimum to the other is not possible.

Table S15: Average $P_\alpha - P_\beta - P_\gamma$ angle values from simulations in solution and the effect of QM/MM structure optimization

			θ $(P_\alpha, P_\beta, P_\gamma)$ / °	$\theta_{MM} - \theta_{QM}$ / °
min. ionic conc.		MM	101.38	4.38
		QM	97.00	
high ionic conc.	single ATP	MM	133.16	4.57
		QM	128.59	
	two ATP	MM	110.70	2.78
		QM	107.92	

12 Changes in the $P_\alpha - P_\beta - P_\gamma$ angle of ATP and ADP.P$_\mathrm{i}$ inside the binding pocket of p97

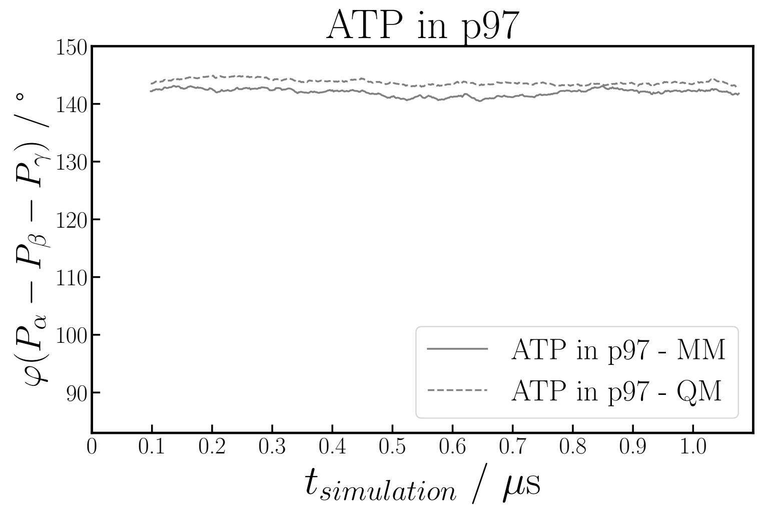

Figure S20: Time evolution of $P_\alpha - P_\beta - P_\gamma$ angles in ATP bound to p97. For the rolling averages a window size of 50 frames was used.

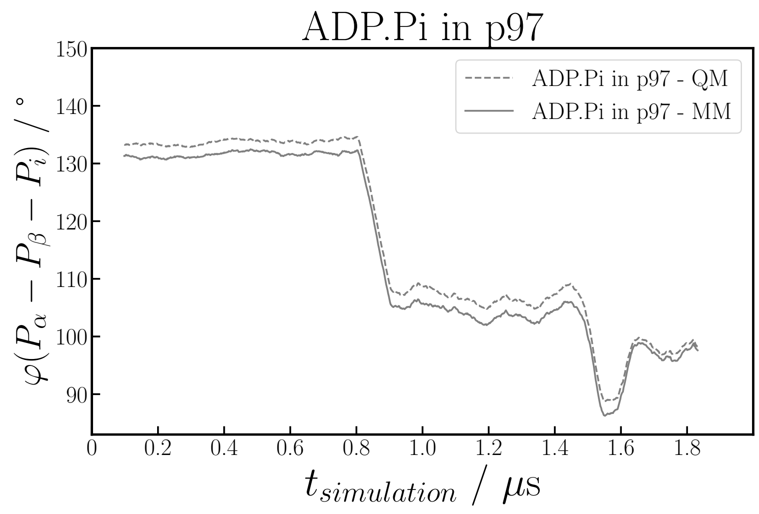

Figure S21: Time evolution of $P_\alpha - P_\beta - P_\gamma$ angles in ADP.P$_\mathrm{i}$ bound to p97. For the rolling averages a window size of 50 frames was used.

We examined the importance of various geometrical features inside the protein and observed that the $P_\alpha - P_\beta - P_\mathrm{i}$ angle remains significant and has an impact on chemical shifts even after the cleavage of the P_γ (see Figure 11 - main manuscript). The trend in predicted chemical shifts for ADP.P$_\mathrm{i}$ over the simulation time closely resembles the change in the $P_\alpha - P_\beta - P_i$ angle.

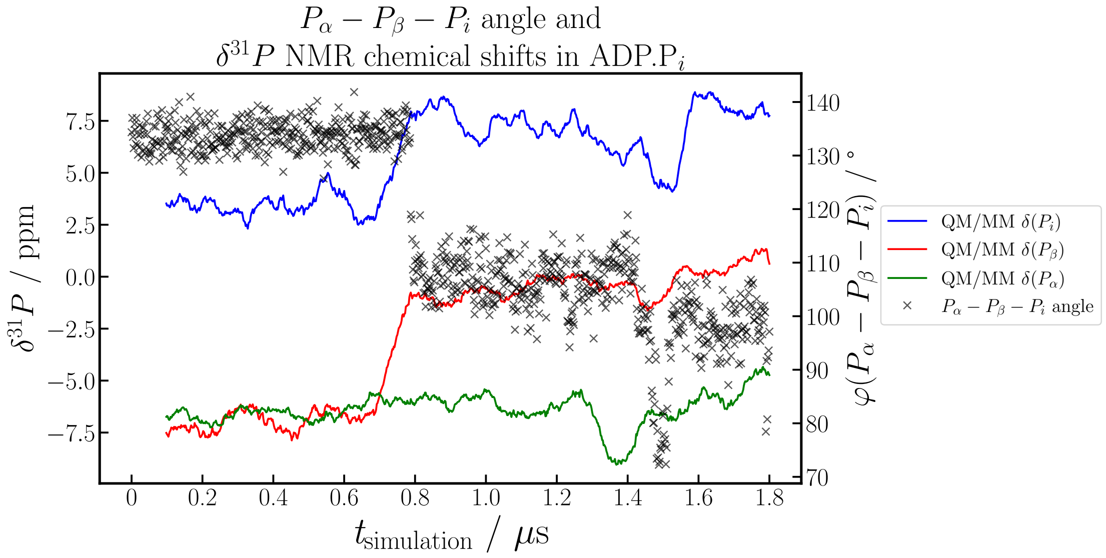

Figure S22: Time evolution of the $P_\alpha - P_\beta - P_\mathrm{i}$ angles and computed chemical shifts in ADP.P$_\mathrm{i}$ bound to p97. For the rolling averages a window size of 30 snapshots was used.

13 Feature importance analysis with logistic regression

The 'LogisticRegression' module from the Scikit-Learn Python library[17] was used for logistic regression analysis. The data set was divided into a training set and a test set, with 20% of the data allocated to the test set and 80% to the training set. Features with higher variance (dihedrals, angles) might dominate the learning process, therefore our dataset was standardized by removing the mean and scaling to unit variance. We used both L1 and L2 regularization to assess the influence and significance of individual geometrical features. To ensure robustness and reliability of the results, repetitions were included by repeatedly splitting the data, fitting the model and computing the feature importance scores 100 consecutive times with different random states. The 'saga' solver algorithm was used in the optimization problem. In order to prevent overfitting in the machine learning model, a strong regularization was used by setting the strength to 0.2.

14 Correlation analysis for the selected structural features in ADP.P_i

The selected structural features' correlation (see Figure 11 - main text) was analyzed using the Pearson correlation coefficient. The dataset contains the measured values for distances, dihedrals, and angles from all snapshots of the ADP.P_i trajectory in p97. Raw angles have a periodic behavior, rendering them unsuitable for correlation coefficients that assume a linear relationship between variables. To address this, when computing the Pearson correlation coefficient for dihedrals (θ) and angles (φ), we employed cosine-transformed values instead of the raw data.

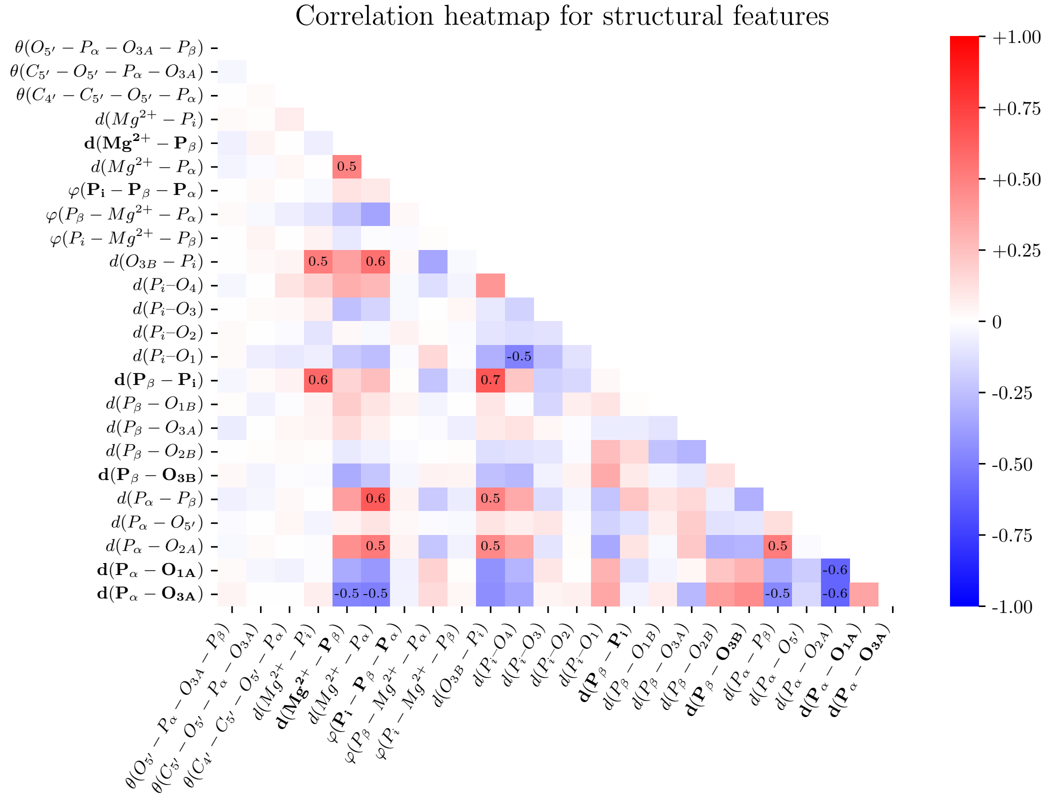

Figure S23: Pearson correlation heatmap for structural features used in the feature importance analysis (Figure 11 - main text) - the labels of the most important features are highlighted with bold. The correlation coefficient ranges from -1 (blue) to +1 (red). Values for coefficients, where the absolute values are below 0.5 are not shown on the heatmap.

These structural variables are not completely independent of each other but no strong correlation is found among the most important features: $\varphi(P_i - P_\beta - P_\alpha)$, $d(P_\beta - O_{3B})$, $d(P_\alpha - O_{3A})$, $d(P_\alpha - O_{1A})$, $d(P_\beta - P_i)$, $d(Mg^{2+} - P_\beta)$.

References

(1) Phillips, J. C.; Hardy, D. J.; Maia, J. D.; Stone, J. E.; Ribeiro, J. V.; Bernardi, R. C.; Buch, R.; Fiorin, G.; Hénin, J.; Jiang, W.; McGreevy, R.; Melo, M. C.; Radak, B. K.; Skeel, R. D.; Singharoy, A.; Wang, Y.; Roux, B.; Aksimentiev, A.; Luthey-Schulten, Z.; Kalé, L. V.; Schulten, K.; Chipot, C.; Tajkhorshid, E. Scalable molecular dynamics on CPU and GPU architectures with NAMD. *J. Chem. Phys.* **2020**, *153*, 044130.

(2) Meagher, K. L.; Redman, L. T.; Carlson, H. A. Development of polyphosphate parameters for use with the AMBER force field. *J. Comput. Chem.* **2003**, *24*, 1016–1025.

(3) Jorgensen, W. L.; Chandrasekhar, J.; Madura, J. D.; Impey, R. W.; Klein, M. L. Comparison of simple potential functions for simulating liquid water. *J. Chem. Phys.* **1983**, *79*, 926–935.

(4) Ryckaert, J.-P.; Ciccotti, G.; Berendsen, H. J. C. Numerical integration of the cartesian equations of motion of a system with constraints: molecular dynamics of n-alkanes. *J. Comput. Phys.* **1977**, *23*, 327–341.

(5) Jaffe, E. K.; Cohn, M. ^{31}P nuclear magnetic resonance spectra of the thiophosphate analogs of adenine nucleotides; effects of pH and Mg^{2+} binding. *Biochemistry* **1978**, *17*, 652–657.

(6) Kästner, J.; Carr, J. M.; Keal, T. W.; Thiel, W.; Wander, A.; Sherwood, P. DL-FIND: an open-source geometry optimizer for atomistic simulations. *J. Phys. Chem. A* **2009**, *113*, 11856–11865.

(7) Lu, Y.; Farrow, M. R.; Fayon, P.; Logsdail, A. J.; Sokol, A. A.; Catlow, C. R. A.; Sherwood, P.; Keal, T. W. Open-Source, python-based redevelopment of the ChemShell multiscale QM/MM environment. *J. Chem. Theory Comput.* **2018**, *15*, 1317–1328.

(8) Wang, L.-P.; Song, C. Geometry optimization made simple with translation and rotation coordinates. *J. Chem. Phys.* **2016**, *144*.

(9) Kussmann, J.; Luenser, A.; Beer, M.; Ochsenfeld, C. A reduced-scaling density matrix-based method for the computation of the vibrational Hessian matrix at the self-consistent field level. *J. Chem. Phys.* **2015**, *142*, 094101.

(10) Kussmann, J.; Ochsenfeld, C. Pre-selective screening for matrix elements in linear-scaling exact exchange calculations. *J. Chem. Phys.* **2013**, *138*, 134114.

(11) Kussmann, J.; Ochsenfeld, C. Preselective screening for linear-scaling exact exchange-gradient calculations for graphics processing units and general strong-scaling massively parallel calculations. *J. Chem. Theory Comput.* **2015**, *11*, 918–922.

(12) Kussmann, J.; Ochsenfeld, C. Hybrid CPU/GPU integral engine for strong-scaling ab initio methods. *J. Chem. Theory Comput.* **2017**, *13*, 3153–3159.

(13) Ochsenfeld, C.; Kussmann, J.; Koziol, F. Ab initio NMR spectra for molecular systems with a thousand and more atoms: a linear-scaling method. *Angew. Chem., Int. Ed.* **2004**, *116*, 4585–4589.

(14) Wilson, P. J.; Bradley, T. J.; Tozer, D. J. Hybrid exchange-correlation functional determined from thermochemical data and ab initio potentials. *J. Chem. Phys.* **2001**, *115*, 9233–9242.

(15) Jensen, F. Segmented contracted basis sets optimized for nuclear magnetic shielding. *J. Chem. Theory Comput.* **2015**, *11*, 132–138.

(16) Buelens, F. P.; Leonov, H.; de Groot, B. L.; Grubmüller, H. ATP–Magnesium Coordination: Protein Structure-Based Force Field Evaluation and Corrections. *J. Chem. Theory Comput.* **2021**, *17*, 1922–1930.

(17) Pedregosa, F.; Varoquaux, G.; Gramfort, A.; Michel, V.; Thirion, B.; Grisel, O.; Blondel, M.; Prettenhofer, P.; Weiss, R.; Dubourg, V.; Vanderplas, J.; Passos, A.; Cournapeau, D.; Brucher, M.; Perrot, M.; Duchesnay, E. Scikit-learn: Machine Learning in Python. *J. Mach. Learn. Res.* **2011**, *12*, 2825–2830.
